# Reduced expression of UPF1 promotes tumor progression through stabilizing COX-2 mRNA in nasopharyngeal carcinoma

**DOI:** 10.3389/fimmu.2025.1617864

**Published:** 2025-11-10

**Authors:** Chun Wu, Ruowen Xiao, Lin Zhang, Zixuan Chen, Meiyin Zhang, Jiaxin Li, Yufeng Zhou, Shuocheng Wang, Chao Jiang, Huiyun Wang, Rui Sun, Shijuan Mai

**Affiliations:** 1 State Key Laboratory of Oncology in South China, Guangdong Key Laboratory of Nasopharyngeal Carcinoma Diagnosis and Therapy, Guangdong Provincial Clinical Research Center for Cancer, Sun Yat-sen University Cancer Center, Guangzhou, China; 2 Department of Oncology, The First Hospital of Lanzhou University, Lanzhou, Gansu, China; 3 Department of Clinical Laboratory, Sun Yat-Sen University Cancer Center, Guangzhou, China; 4 Department of Nasopharyngeal Carcinoma, Sun Yat-sen University Cancer Center, Guangzhou, China; 5 Department of Cancer Center, The People’s Hospital of Shenzhen Baoan District, The Second Affiliated Hospital of Shenzhen University, Shenzhen, Guangdong, China

**Keywords:** up-frameshift 1, nonsense-mediated mRNA decay, nasopharyngeal carcinoma, cyclooxygenase-2, macrophage

## Abstract

**Background:**

UPF1 (upframeshift 1) is the core factor in the nonsense-mediated mRNA decay (NMD) pathway. UPF1 is dysregulated in multiple human malignancies and may play a role in cancer progression. However, the expression level and function of UPF1 in nasopharyngeal carcinoma (NPC) have remained undocumented until now.

**Methods:**

UPF1 expression in NPC tissues was evaluated by using qRT–PCR and immunohistochemistry assays. *In vitro* and *in vivo* experiments were performed to examine the effects of UPF1 on NPC cells. NMD targets were identified by RNA-seq and RNA stability analysis. Rescue experiments were employed to reveal the underlying molecular mechanisms that mediate the tumor suppressive role of UPF1. The effects of UPF1 knockdown NPC cells on macrophages and T cells were detected by using an indirect coculture system.

**Results:**

UPF1 expression was significantly downregulated in NPC tissues and correlated with a poor prognosis. UPF1 overexpression inhibited NPC cell growth and metastasis both *in vitro* and *in vivo*. COX-2 and PD-L1 were identified as the key targets of UPF1-mediated NMD in NPC. Reduced expression of UPF1 activated the ERK/MAPK and JAK2/STAT3 pathways and enhanced NPC cell viability through upregulation of COX-2. Moreover, coculture with UPF1-knockdown NPC cells promoted macrophage M2 polarization and migration and suppressed CD8^+^ T-cell activation.

**Conclusions:**

Our findings suggest that reduced expression of UPF1 in NPC might contribute to tumor progression.

## 1 Introduction

Nonsense-mediated mRNA decay (NMD) is a highly conserved pathway in eukaryotic cells that regulates the quality and abundance of cellular transcripts. Initially, NMD was viewed as a cellular surveillance process to selectively degrade erroneous RNAs harboring truncating mutations that prematurely terminate translation, which can be created by nonsense mutations, frameshift mutations and aberrant RNA splicing. It is estimated that NMD degrades up to 30% of all mutated protein-coding mRNAs (1, 2). However, with the progress of research, it was found that about 10% of human wild-type coding physiological genes are also degraded by NMD (3). NMD substrates have been implicated in a diverse range of biological processes, including development, the cell cycle, cell viability, differentiation, cellular stress and immune responses (4–6). NMD is suppressed in response to translation initiation factor eIF2α phosphorylation during ER stress; thus, disturbances in NMD efficiency are frequently observed in various cancers. Impaired NMD leads to increased stability of NMD target genes; accordingly, significant enrichment of NMD target mRNAs was detected in tumorigenic breast, colon, liver, lung, thyroid, and esophageal tissues compared with normal tissues, whereas no significant difference in the expression levels of non-NMD targets was detected (7).

UPF1 (upframeshift 1) is considered the most important regulator of the NMD pathway. Dysregulation of UPF1 has been reported in multiple cancers (8–10). UPF1 knockdown contributes to the progression of hepatocellular carcinomas through the targeting of Smad7 (10). Similarly, reduced UPF1 expression in lung adenocarcinoma led to NMD suppression, which activated the TGF-β signaling pathway and contributed to cancer metastasis (11). In breast cancer, NMD may have anticancer effects through the degradation of mutant BRCA1 mRNAs encoding a truncated protein with a tumor-promoting dominant negative effect (12). A tumor suppressive role of UPF1 was also observed in tumor cell lines, including gastric cancer (11), ovarian cancer (13) and glioma (14) cells.

To date, the expression and function of NMD factors in nasopharyngeal carcinoma (NPC) have not been studied. In the present study, we revealed that UPF1 mRNA and protein expression levels were significantly lower in NPC tissues than in nontumor tissues and correlated with a poor patient prognosis. Overexpression of UPF1 suppressed NPC cell proliferation and metastasis *in vitro* and *in vivo*. RNA-sequencing (RNA-seq) analysis suggested that UPF1 expression was correlated with immune-related pathways, and COX-2 and PD-L1 were identified as potential targets of UPF1 in NPC. Reduced UPF1 promoted NPC cell growth through upregulation of COX-2 and activation of the ERK/MAPK and JAK2/STAT3 pathways. Moreover, coculture with UPF1-knockdown NPC cells increased M2 macrophage polarization and reduced the ratio of activated CD8^+^ T cells. The results of our study suggest that decreased UPF1 expression in NPC cells might enhance tumor progression.

## 2 Materials and methods

### 2.1 Cell lines, reagents and antibodies

HK1, 5-8F and 6-10B human NPC cells were preserved at the State Key Laboratory of Oncology in South China and routinely maintained in RPMI 1640 medium (Gibco, Canada) supplemented with 10% fetal bovine serum (FBS) (Sigma, USA) at 37 °C in a 5% CO_2_ humidified atmosphere. 5-8F (high metastatic) and 6-10B (low metastatic) are subclones derived from the NPC cell line SUNE1. Human monocytic THP-1 cells were grown in suspension in RPMI 1640 supplemented with 10% FBS (Sigma, #F8318). All the cell lines were authenticated using short tandem repeat profiling and tested for Mycoplasma contamination.

The commonly used chemical inhibitor of NMD, Emetine, was purchased from MCE (HY-B1479B, MCE, China). The selective COX-2 inhibitor celecoxib (SC 58635), p38 MAPK inhibitor SB203580 (S1076) and STAT3 inhibitor cryptotanshinone (S2285) were purchased from Selleck (Selleck Chemicals, Houston, TX, USA). The human CD3/CD28/CD2 T-cell activator was obtained from Stemcell (STEMCELL™ Technologies, Canada).

Rabbit monoclonal antibodies against human UPF1 and PD-L1 were purchased from Abcam (Cambridge, UK). Rabbit monoclonal antibodies against human COX-2, p-JAK2 (Tyr1007/1008), STAT3, p-STAT3 (Tyr705), ERK1/2, p-ERK1/2 (Thr202/Tyr204), p38 MAPK, p-p38 MAPK (Thr180/Tyr182), AKT, p-AKT (Ser473), mTOR, p-mTOR (Ser2448), NF-κB, p-NF-κB (Ser536) and GAPDH were purchased from Cell Signaling Technology (Danvers, MA, USA). The surface antibodies against CD8, CD69, CD107A, CD206 and CD86 used for flow cytometry were obtained from BD Biosciences (USA). The antibodies against human COX-2 and PD-L1 used for ELISA detection were purchased from MyBioSource (USA). The antibodies against human IFN-γ, Granzyme B (GZMB) and IL-2 used for ELISA detection were purchased from ABclonal Technology Co., Ltd (Wuhan, China).

### 2.2 Clinical samples

Twenty-two samples of snap-frozen NPC tissues and 14 noncancerous nasopharyngeal tissues were obtained at the time of diagnosis at Sun Yat-Sen University Cancer Center (SYSUCC) and preserved in RNAlater (Ambion, Inc., USA) at -80 °C before being used for quantitative real-time PCR (qRT–PCR) detection. Formalin-fixed, paraffin-embedded tissues from 67 primary NPC tissues were obtained from the archives of the Department of Pathology at SYSUCC between January 2007 and December 2008. All patients were histologically and clinically diagnosed with NPC. None of the patients received radiotherapy or chemoradiotherapy before biopsy. This study was approved by the Research Ethics Committee of SYSUCC, and written informed consent was obtained from each participant prior to the research.

### 2.3 Vectors and transfection

Lentiviruses overexpressing UPF1 were purchased from GenePharma (Shanghai, China). NPC cells were infected with recombinant lentivirus plus 8 μg/ml polybrene (Sigma, St Louis, Missouri, USA). Stable cell lines were selected using 4 μg/mL puromycin. Transient transfection was performed using Lipofectamine 2000 (Invitrogen, CA, USA) in OPTI-MEM. siRNAs against UPF1 and their negative controls were obtained from RiboBio (Guangzhou, China).

### 2.4 NMD activity measurement

NMD efficiency was measured by using the NMD reporter plasmid pTRE-Tight-BI-Gl NORM-LacZA-TER-LacZB, a gift from Robert Singer (Addgene plasmid # 86194; http://n2t.net/addgene:86194; RRID: Addgene_86194). PTC-free β-globin mRNA (termed Gl NORM mRNA) that is resistant to NMD and PTC-containing β-globin mRNA (termed Gl TER mRNA) that is subjected to NMD were coexpressed from a doxycycline (Dox)-inducible bidirectional promoter in this single plasmid, and the ratio of the TER/NORM mRNA expression level indicates the efficiency of NMD. As described previously (15), the cells were seeded in six-well plates (1 × 10^5^ cells/well) and cultured with RPMI 1640/10% FBS overnight. Then, the cells were transiently transfected with the pTRE-Tight-BI-Gl NORM-LacZA-TER-LacZB plasmid, and 1 μg/ml doxycycline (MedChemExpress, HY-N0565B) was subsequently added to the medium. After 16 h, the medium was removed and replaced with complete medium supplemented with 4 μg/mL actinomycin D (ActD; MedChemExpress, HY-17559) to inhibit transcription. RNA was harvested at the indicated time points, and NORM LacZA and TER LacZB mRNA levels were measured using qRT–PCR.

### 2.5 NMD inhibition

The inhibition of the NMD pathway was performed according to a previously described protocol (16). Briefly, the cells were incubated for 10 h in complete culture medium supplemented with 100 μg/ml of Emetine dihydrochloride hydrate (MedChemExpress, HY-B1479B) before total RNA was extracted. Mock-treated cells were used as a reference.

### 2.6 mRNA stability assay

To measure RNA degradation targeted by NMD, cells in serum-free DMEM were incubated with 10 μg/ml cycloheximide (GLPBIO, GC17198-1) for 16 hours to inhibit translation-dependent mRNA decay (17). The cells were subsequently allowed to recover for 4 hours in serum-free DMEM before 10 μg/ml actinomycin D was added to inhibit transcription. After the indicated times, the total RNA was harvested and analyzed by real-time PCR.

### 2.7 RNA extraction and qRT-PCR

Total RNA was extracted with TRIzol (Sigma–Aldrich, St Louis, MO, USA) according to the manufacturer’s instructions. cDNA was synthesized with a cDNA reverse transcription kit (Thermo Fisher Scientific, MA, USA). Quantitative real-time PCR was carried out using a SYBR Green PCR kit (Thermo Fisher Scientific, MA, USA) as described previously (18). The specific sequences of the primers used in this study are listed in **Supplementary Table 1**.

### 2.8 Western blotting

Western blotting was performed according to the methods described in our previous report (18). Briefly, total protein was extracted from cultured cells using RIPA buffer supplemented with PMSF and quantified using a BCA protein assay kit (Beyotime, Haimen, China). Protein lysates were subjected to SDS–PAGE and transferred onto polyvinylidenedifluoride membranes (Millipore, Billerica, MA), followed by incubation first with a primary antibody and then with a secondary antibody. The signals were detected with a KeyGEN Enhanced ECL detection kit according to the manufacturer′s instructions (KeyGEN, Nanjing, China).

### 2.9 RNA sequencing and data analysis

RNA was extracted from UPF1 knockdown (UPF1-KD), UPF1-overexpressing, and Emetine-treated NPC cells and their corresponding control cell lines using TRIzol Reagent (Thermo Fisher Scientific, MA, USA) according to the manufacturer’s protocol. RNA degradation and contamination were monitored on 1% agarose gels. RNA purity was checked using a NanoPhotometer^®^ spectrophotometer (IMPLEN, CA, USA). The RNA concentration was measured using a Qubit^®^ RNA Assay Kit in a Qubit^®^ 2.0 fluorometer (Life Technologies, CA, USA). A total of 3 μg of RNA per sample was used as input material for the RNA sample preparations. Sequencing libraries were generated using the NEBNext^®^ UltraTM RNA Library Prep Kit for Illumina^®^ (NEB, USA) following the manufacturer’s recommendations, and index codes were added to attribute sequences to each sample. Sequencing was performed using the Illumina NovaSeq 6000 platform (Novogene, Beijing, China).

Three biological replicates were analyzed for UPF1 overexpressing HK1 and HK1-NC cell lines. Considering that the 5-8F and 6-10B cells derived from the same parental line, biological replicates were not used for sequencing of UPF1-KD 6-10B cells, Emetine treated 5-8F cells and their corresponding controls. Raw data (raw reads) of fastq format were firstly processed through in-house perl scripts, yielding clean reads. Index of the reference genome was built using Hisat2 v2.0.5 and paired-end clean reads were aligned to the reference genome using Hisat2 v2.0.5. Gene expression was quantified using featureCounts v1.5.0-p3. FPKM of each gene was calculated based on the length of the gene and reads count mapped to this gene. Differential expression analysis used DESeq2 v1.16.1 (with biological replicates; negative binomial model) or edgeR v3.18.1 (without replicates; count normalization). Genes with fold change > 2 and *P* < 0.05 were assigned as differentially expressed. Differentially expressed genes (DEGs) were analyzed by the KEGG pathway using the WEB-based GEneSeT AnaLysis Toolkit (webgestalt) (https://www.webgestalt.org) based on over-representation analysis (ORA). All genes in the genome were used as the enrichment background. Terms with *P* < 0.05, minimum count = 4, and enrichment factor > 3 were collected and grouped into clusters based on membership similarities.

### 2.10 Immunohistochemistry

IHC was performed using a standard streptavidin–biotin–peroxidase complex method according to our previous report (19). The staining results were evaluated independently by two pathologists. The intensities were graded as 0 (negative), 1 (weakly positive), 2 (moderately positive) and 3 (strongly positive), and the estimated fraction of positively stained tumor cells was evaluated as the percentage from 0 to 100%. Finally, the intensity and proportion score were multiplied to determine the total IHC score of each section.

### 2.11 Multiplex immunohistochemistry

Multiplex immunohistochemistry was carried out using the PANO 7-plex IHC Assay Kit (Panovue, Beijing, China) according to the manufacturer’s protocol. Tissue sections were first incubated at 65°C for 2 hours to improve adhesion to the slides. Deparaffinization was performed in xylene, followed by rehydration through a graded ethanol series (100%, 95%, 70%, and 50%). Prior to antigen retrieval, samples were fixed in 10% neutral buffered formalin for 30 minutes. Antigen unmasking was achieved via microwave-assisted heating in EDTA-based retrieval buffer (pH 9.0; ZSGB-Bio, Beijing, China). After blocking to minimize nonspecific binding, primary antibodies were applied in a sequential manner, each paired with HRP-conjugated secondary antibodies. To enhance signal detection, tyramide signal amplification (TSA) was employed following each primary antibody incubation. Subsequently, sections were incubated with biotinylated rabbit polyclonal antibodies targeting rabbit and mouse IgG, followed by HRP-labeled streptavidin, as outlined in the kit instructions (Panovue, Beijing, China).For detection, chromogenic reactions were developed using biotinylated secondary antibodies in combination with streptavidin-alkaline phosphatase, while fluorescent signals were generated using streptavidin-conjugated fluorophores with excitation wavelengths at 480, 520, 570, 690, and 780 nm. Multispectral imaging was performed using the Vectra Polaris Automated Quantitative Pathology Imaging System (Akoya Biosciences, Delaware, USA) for high-plex spatial analysis.

### 2.12 Enzyme-linked immunosorbent assay

Human COX-2 ELISA Kit and Human CD274 ELISA Kit were purchased from Ruixinbio™ (Shanghai, China). Samples from each group were harvested at 48h post-transfection of siRNAs against UPF1 and centrifuged at 2000 rpm for 20 min at 4 °C to obtain supernatants. COX-2 and PD-L1 levels in cell culture supernatants were quantified according to the manufacturer’s instructions. Based on the standard dilution curve, the optimal detection range was 5.6 ng/mL – 25.6 ng/mL for COX-2, and 0.7 ng/mL – 3.6 ng/mL for PD-L1.

### 2.13 Cell viability assay

Cell viability was determined by a CCK8 Cell Counting Kit (JingXin Biological Technology, Guangzhou, China) (19). Cells were plated and cultured in 96-well plates at a density of 1 × 10^3^ cells/well. The detection was performed at the same time every day. The OD value was measured at 450 nm by a microplate reader (SpectraMax^®^ M5 Multimode Microplate Reader; Molecular Devices, LLC, Sunnyvale, CA, USA). All experiments were performed in triplicate.

### 2.14 Colony formation assay

The protocol used for the clonogenic survival assay was described in our previous study (20). Cells were seeded in six-well plates (300 cells/well) and cultured for 2 weeks. Colonies were fixed with methanol and stained with 0.1% crystal violet in 20% methanol for 15 min. Colonies larger than 0.1 mm in diameter were scored. The experiments were performed in triplicate for each cell line.

### 2.15 Migration and invasion assays

Transwell migration and invasion analyses were carried out as previously described (20). To assess cell migration, 5 × 10^4^ cells were placed into the upper chambers (BD Biosciences, Lexington, UK) with 200 µl serum free medium, whereas the lower chambers in the 24 well plate contained 650 µl complete medium with 10% FBS. After 16 h, the cells were fixed with methanol and stained with 0.1% crystal violet. Cells in the upper chambers were removed gently, and the cells that had migrated into the membranes were imaged under an inverted fluorescence microscope. The number of migratory cells was counted for statistical use.

For invasion assays, 5 × 10^4^ cells were plated in a Matrigel-coated Transwell chamber (BD Biosciences, Lexington, UK) with an 8 µm pore size. The number of invaded cells was counted 48 h later. The experiments were performed in triplicate.

### 2.16 Polarization of THP-1 macrophages

THP-1 monocytes in suspension were treated with phorbol 12-myristate 13-acetate (PMA, 100 ng/ml; Sigma, St. Louis, MO) for 48 hours to allow them to attach and differentiate into intermediate-stage M0 cells. To generate M2-polarized macrophages (M2-THP1), M0 macrophages were exposed to the M2 cytokines IL4 (20 ng/ml; PeproTech, Rocky Hill, NJ) and IL13 (20 ng/ml; PeproTech, Rocky Hill, NJ) for an additional 48 hours (21).

### 2.17 Coculture of NPC cells with macrophages

Indirect coculture experiments were performed using a 24-well Transwell apparatus with a 0.4 µm pore size (Corning, NY, USA) (22). The THP-1 cells were induced into M0 status by treatment with PMA for 48 h, and the M0 cells were then placed in the upper chamber of the Transwell plate at a density of 1 × 10^5^/well, and the lower wells contained UPF1-KD or control NPC cells (1 × 10^5^). Macrophages without coculture were also used as controls. The macrophages were harvested 72 h later and their phenotypic changes were determined by flow cytometry and qRT–PCR.

### 2.18 Macrophage migration assay

To study the recruitment of macrophages by NPC cells, inserts with a membrane pore size of 8 μm (Corning, NY, USA) were used for the Transwell migration assay (23). UPF1-KD or control NPC cells (5 × 10^5^) were placed in the lower compartment of a 24-well plate, and after 24 hours, M2 macrophages (1 × 10^6^) were added to the upper chamber and incubated at 37°C and 5% CO_2_ for 36 hours. The rescue experiments were performed by knocking down COX-2 expression in NPC cells with siRNAs against COX-2 before they were cocultured with macrophages. The number of migrated M2 macrophages was quantified using ImageJ from images captured with an inverted microscope.

### 2.19 Flow cytometry

After being coincubated with UPF1-KD or control NPC cells for 72 h in six-well plates, the PBMCs were stained with anti-CD8-FITC (BD Biosciences, San Jose, CA, USA), anti-CD69-PC7 (BD Biosciences, San Jose, CA, USA) and anti-CD107A-BV450 (BD Biosciences, San Jose, CA, USA). Polarized THP-1 cells were stained with anti-CD86-APC (BD Biosciences, San Jose, CA, USA) and anti-CD206-PE (BD Biosciences, San Jose, CA, USA) after they were cocultured with UPF1-KD or control NPC cells for 72 hours. Flow cytometry analysis was carried out as previously described (21). The results were analyzed using FlowJo 10.7 software (BD Biosciences, USA).

### 2.20 T cell proliferation assay

PBMCs were isolated from peripheral venous blood from healthy donors and stimulated using anti-CD3/anti-CD28 antibodies (StemCell Technologies, Vancouver, CA) and IL2 (PeproTech, Rocky Hill, NJ) (24). After being labeled with carboxyfluorescein succinimidyl ester (CFSE) (C34554; Thermo Fisher Scientific, USA), preactivated T cells were cocultured with NPC cells infected with UPF1-KD lentivirus or control lentivirus in six-well plates for 72 h, and the CFSE density was subsequently measured by flow cytometry. Cell surface phenotyping was performed by staining with anti-CD8-FITC (BD Biosciences, San Jose, CA, USA), anti-CD69-PC7 (BD Biosciences, San Jose, CA, USA) or anti-CD107A-BV450 (BD Biosciences, San Jose, CA, USA) antibodies prior to flow cytometry analysis. The results were analyzed using the FlowJo 10.7 software program.

### 2.21 Animal study

Six-week-old male athymic nude mice were injected subcutaneously with 6 × 10^6^ 5-8F-UPF1 or 5-8F-control cells (n = 8/group). The resulting tumors were examined every 3 days. After 30 days, the mice were killed, and the subcutaneous tumors were resected. Tumor size was measured using calipers, and tumor volume was calculated (V = 0.5 × L ×W^2^). UPF1 expression levels in the subcutaneous tumors were confirmed by western blot analysis.

Four-week-old male nude mice (n = 7/group) were subcutaneously injected with 6 × 10^6^ UPF1-knockout or control HK1 cells, respectively. Twenty-one days post-injection, the mice were euthanized, and subcutaneous tumors were excised. Tumor masses were weighed, and UPF1 expression was confirmed via multiplex immunohistochemistry (IHC).

For the *in vivo* metastasis assays, fourteen 3- to 4-week-old male BALB/c-nu mice were randomized into 2 groups of 6 mice each. Afterward, 100 μl of cell suspension containing 1 × 10^6^ 5-8F-UPF1 or control cells was injected intravenously through the tail vein into each mouse. The experiment was terminated after 8 weeks, and the metastatic nodules in the lungs were carefully examined and counted as described previously (19). Eighteen 3- to 4-week-old male BALB/c-nu mice were randomly assigned to two groups (n = 9/group). Each mouse was injected intracardially into the left ventricle with 100 μL of cell suspension containing 1 × 10^5^ UPF1-knockout or control HK1 cells. The experiment was terminated three weeks post-injection, and metastatic lung nodules were carefully examined and quantified.

The animals were housed under standard conditions and cared for according to the institutional guidelines for animal care. All animal experiments in this study were performed in accordance with the guidelines of the Animal Ethics Committee of Sun Yat-sen University (approval no. L102012017004Y) on February 20, 2017.

### 2.22 Statistical analysis

The sample size was chosen on the basis of the need for statistical power. All experiments were repeated three times or more, and the data are presented as the mean ± SD. Student’s *t* test (unpaired, two-tailed) was used to evaluate significant differences between groups of experimental data where appropriate. The *chi*-square test was used to calculate the probability value for the relationship between two variables using a two-by-two cross-tablulation. Pearson’s correlation analysis was used to evaluate the linear relationship between two variables. The SPSS version 17.0 statistical software package and GraphPad Prism 9 were used for statistical analyses. *P* < 0.05 was considered to indicate statistical significance (*, *P* < 0.05; **, *P* < 0.01; ***, *P* < 0.001).

## 3 Results

### 3.1 UPF1 is frequently downregulated in NPC tissues and cell lines

**Figure 1:**
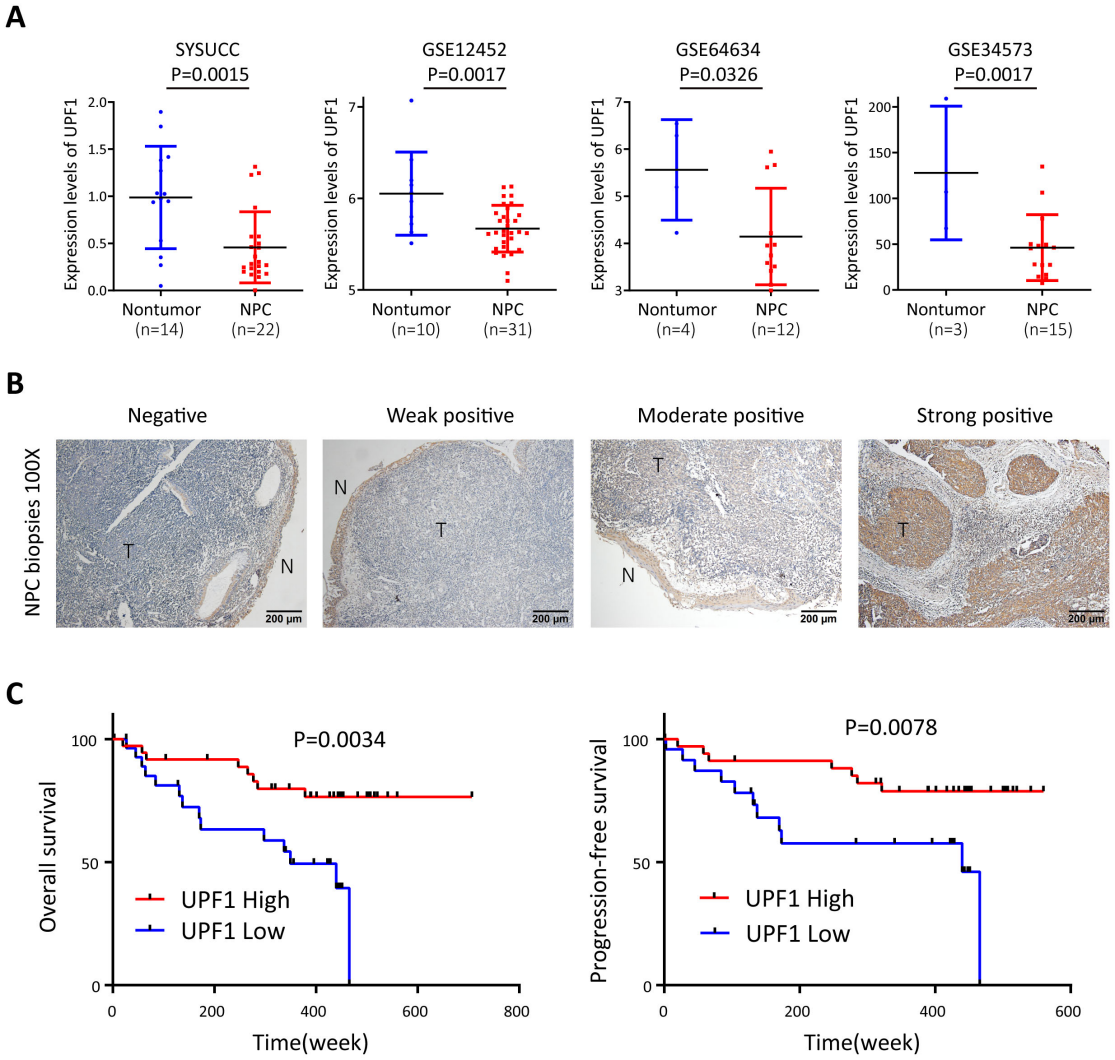
UPF1 is expressed at low levels in NPC and is associated with a poor prognosis of NPC patients. **(A)** Reduced UPF1 expression in 22 NPC tissue samples compared with 14 noncancerous nasopharyngeal epithelial tissue samples derived from SYSUCC was confirmed by qRT–PCR (*P* = 0.0015), and similar results were obtained by analyzing the expression profiles from GSE12452, GSE64634 and GSE34573 in the GEO database. P values were calculated using Student’s *t* tests. **(B)** Representative immunohistochemical staining of UPF1 in NPC tissue sections. Magnification × 100; scale bar, 200 μm. **(C)** Kaplan–Meier survival analysis for overall survival (left) and progression-free survival (right) of 67 patients with NPC with different UPF1 expression levels as determined by IHC.

The expression level of UPF1 was evaluated by qRT–PCR in 22 snap-frozen NPC tissues and 14 noncancerous nasopharyngeal tissues derived from Sun Yat-Sen University Cancer Center, and the results revealed that UPF1 expression was significantly downregulated in tumor tissues compared with that in nontumor controls (**Figure 1A**). Low levels of UPF1 in NPC were further confirmed in the Gene Expression Omnibus (GEO) GSE12452, GSE64634 and GSE34573 datasets (**Figure 1A**). Furthermore, immunohistochemistry (IHC) was performed to measure the protein expression of UPF1 in 67 formalin-fixed, paraffin-embedded NPC samples. UPF1 antibody staining was obviously weaker in the tumor areas than in the adjacent nonmalignant epithelia in most NPC biopsy samples (**Figure 1B**). The optimal threshold value of the IHC score was determined by receiver operating characteristic (ROC) analysis to split the low (31/67, 46.27%) and high UPF1 expression (36/67, 53.73%) groups. Survival analysis revealed that patients with low UPF1 expression had significantly shorter overall survival (OS) and progression-free survival (PFS) than those with high UPF1 expression did (**Figure 1C**).

### 3.2 UPF1 suppressed NPC cell growth and metastasis *in vitro* and *in vivo*

**Figure 2:**
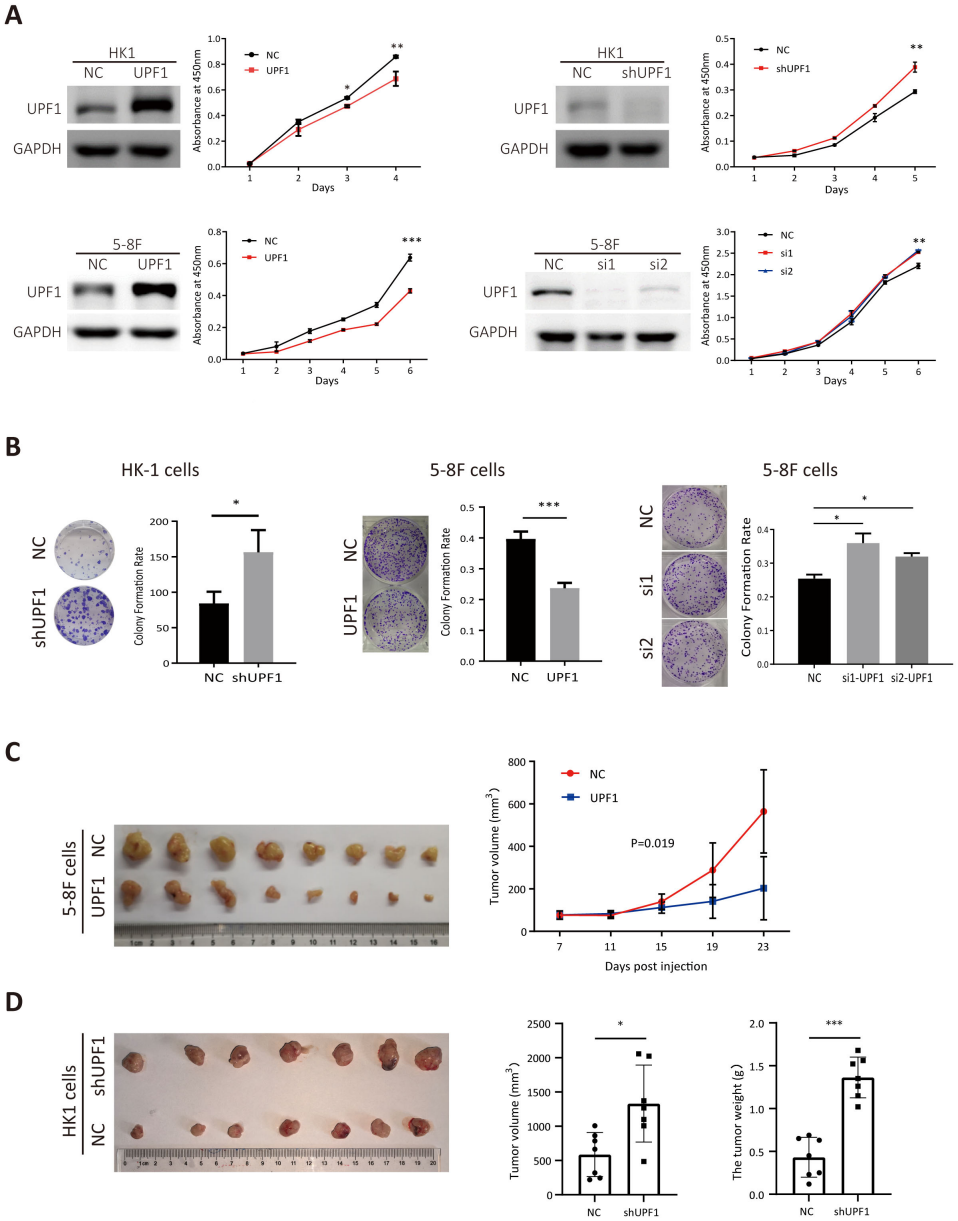
UPF1 inhibits NPC cell growth *in vitro* and *in vivo*. **(A)** Cell growth was evaluated with CCK-8 assays. Western blot analysis was performed to verify the expression levels of UPF1 in NPC cells after transfection of recombinant lentivirus encoding UPF1 or shRNA targeting UPF1 or transient transfection of siRNAs against UPF1. **(B)** Representative images and numbers of colonies formed by UPF1-overexpressing or UPF1-knockdown NPC cells. **(C)** Left, subcutaneous xenograft tumor images for each group were captured 23 days after 5-8F-NC or 5-8F-UPF1 cell injection into nude mice. Right, tumor growth curve, n = 8/group. *P* values were calculated using two-way ANOVA. *P* = 0.019. **(D)** Left, subcutaneous xenograft tumor images for each group were captured 21 days after HK1-NC or HK1-UPF1-KD cell injection into nude mice. Right, tumor weight and volume evaluation in two groups, n = 7/group. *, P < 0.05; **, P < 0.01; ***, P < 0.001.

Stably UPF1-expressing HK1 and 5-8F cells were constructed using lentiviral transfection, and endogenous UPF1 was transiently knocked down in HK1, 5-8F and 6-10B cells with small interfering RNAs (siRNAs) targeting UPF1 or stably depleted by short hairpin RNA (shRNA) against UPF1. UPF1 expression in each cell line was validated by western blotting (**Figure 2A**). The results of the CCK8 and colony formation assays revealed that compared with the control, UPF1 knockdown significantly accelerated cellular proliferation, whereas UPF1 overexpression retarded the proliferation of NPC cells (**Figures 2A, B**).

To investigate whether UPF1 affects tumor growth *in vivo*, 5-8F cells stably overexpressing UPF1 or HK1 cell with stable UPF1 knockdown, along with their corresponding control cells, were inoculated subcutaneously into nude mice. UPF1 expression in subcutaneous tumor tissues was verified using multiplex immunohistochemistry (mIHC) (**Supplementary Figure 1**). As shown in **Figures 2C, D**, UPF1 overexpression led to a marked reduction in tumor volume compared to the control group, whereas UPF1 knockdown significantly enhanced tumor growth *in vivo*.

**Figure 3:**
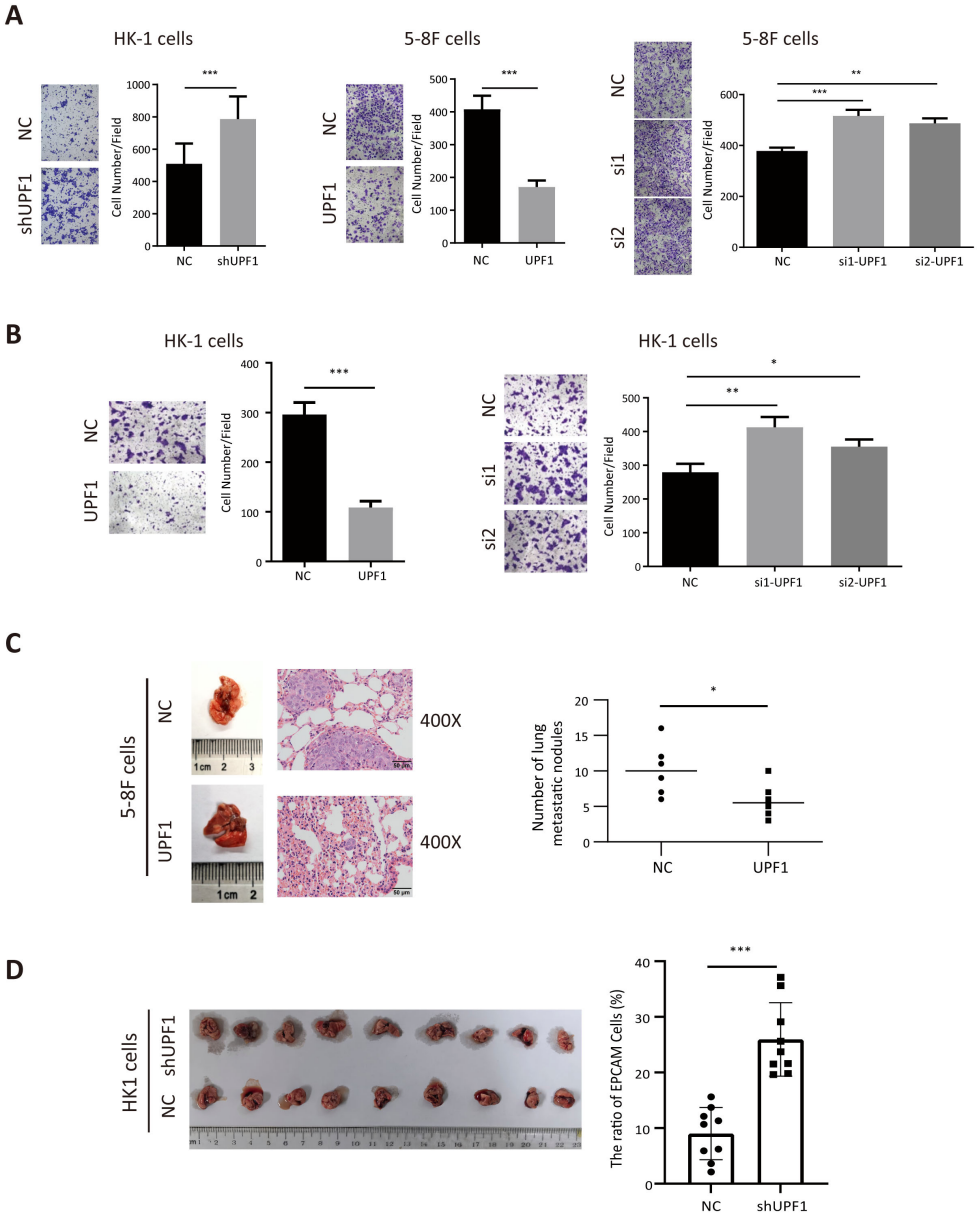
UPF1 inhibits NPC cell metastasis *in vitro* and *in vivo*. **(A)** Representative images and quantitative data of migratory NPC cells after UPF1 overexpression or knockdown. **(B)** Cell invasion ability of UPF1-overexpressing or UPF1-knockdown HK1 cells was measured by Transwell assays with Matrigel. **(C)** 5-8F cells, with or without UPF1 overexpression, were injected into each mouse via the tail vein (n =6/group) to construct lung metastasis models. Representative images (left) and quantification (right) of lung metastasis nodules stained with hematoxylin and eosin from each group (magnification, × 400). **(D)** BALB/c-nu mice were injected intracardially UPF1-KD or control HK1 cells. Three weeks later, the metastatic lung nodules were carefully examined and quantified. *P* values were calculated using two-way ANOVA or Student’s t tests. Data are presented as the mean ± SD. *P < 0.05; **P < 0.01; ***P < 0.001. The experiments were repeated independently at least three times.

Next, the results of the Transwell assays revealed that migratory (**Figure 3A**) and invasion (**Figure 3B**) abilities were significantly decreased in UPF1-overexpressing NPC cells but enhanced in UPF1-KD cells compared with those of the controls. Similar results were observed in 6-10B and S-18 cells (**Supplementary Figure 2**). To further evaluate the effect of UPF1 on metastasis *in vivo*, we employed experimental metastasis assays in nude mice using 5-8F cells stably overexpressing UPF1 or HK1 cell with stable UPF1 knockdown, along with their corresponding control cells. The results showed that mice injected with UPF1-overexpressing 5-8F cells developed significantly fewer metastatic lung nodules compared to the control group (**Figure 3C**). Conversely, mice injected with UPF1-knockdown HK1 cells exhibited a higher number of metastatic nodules relative to controls (**Figure 3D**).

Taken together, these data suggest that UPF1 inhibits the proliferation and metastasis of NPC cells both *in vitro* and *in vivo*.

### 3.3 Pathway analysis and prediction of targets of UPF1 in NPC by RNA-seq analysis

**Figure 4:**
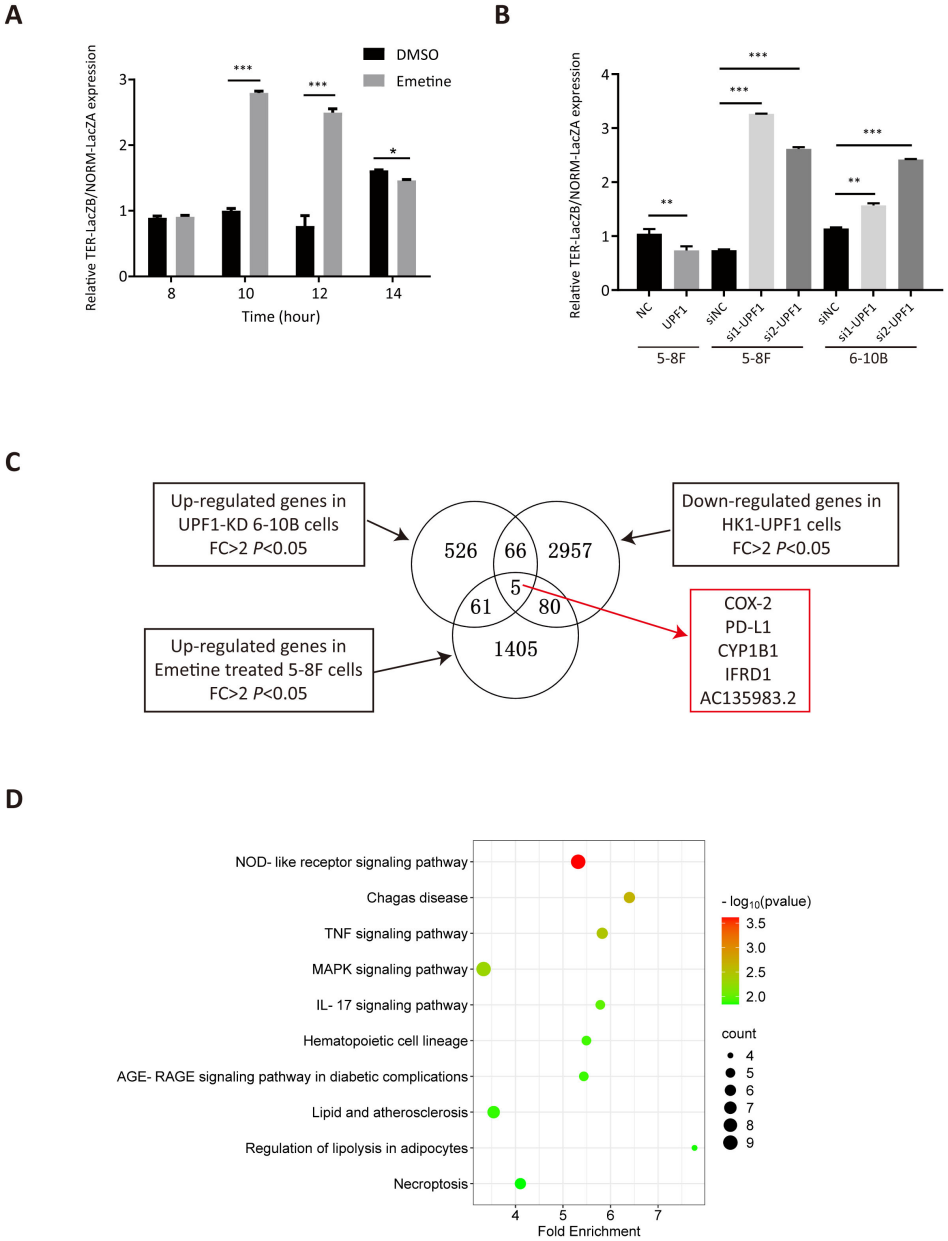
Analysis of differentially expressed genes (DEGs) by RNA-seq. **(A)** NMD activity was inhibited after treatment with Emetine, as validated by the increased TER-LacZB/NORM-LacZA mRNA expression ratio determined by qRT–PCR analysis after transient transfection of the pTRE-Tight-BI-Gl NORM-LacZA-TER-LacZB plasmid. *, P < 0.05; **, P < 0.01; ***, P < 0.001. **(B)** NMD activity was increased in UPF1-overexpressing NPC cells but decreased in UPF1-KD cells, as confirmed by the ratio of TER/NORM mRNA expression levels determined by qRT–PCR analysis. **(C)** RNA-seq was performed using UPF1-overexpressing HK1 cells, 6-10B cells with stable UPF1 knockdown, and Emetine-treated 5-8F cells and their corresponding controls. The overlapping DEGs in the three groups identified by RNA-seq are shown in the Venn diagram. **(D)** Top 10 enriched KEGG pathways of 212 DEGs by WebGestalt based on overrepresentation analysis (ORA), ranked by *P* value.

Chemical translation inhibitors, such as Emetine, can block NMD-mediated degradation. To verify the inhibitory effect of Emetine on NMD activity, we treated 5-8F cells with 100 μg/ml Emetine after transfection of the NMD reporter plasmid pTRE-Tight-BI-Gl NORM-LacZA-TER-LacZB. qRT–PCR was used to quantify the levels of PTC-containing β-Gl TER mRNA and PTC-free β-Gl NORM mRNA relative to those of GAPDH mRNA. Consistent with the findings of previous studies (16), the TER/NORM mRNA expression ratio dramatically increased at 10 h after Emetine treatment, which confirmed that Emetine could reduce NMD efficiency (**Figure 4A**).

To validate the effect of UPF1 on NMD efficiency, UPF1-overexpressing or UPF1-knockdown NPC cells were transiently transfected with the pTRE-Tight-BI-Gl NORM-LacZA-TER-LacZB plasmid and collected for qRT–PCR detection at 2 h after transfection. The results revealed that the TER/NORM mRNA ratio was decreased by UPF1 overexpression and increased by UPF1 inhibition (**Figure 4B**), which confirmed that UPF1 is a key factor in the NMD pathway.

To investigate the UPF1 target genes and their involvement in molecular pathways, RNA-sequencing analysis was performed using HK1 cells with UPF1 overexpression, 6-10B cells in which UPF1 was stably knocked down by UPF1-targeted shRNA transfection, and Emetine-treated 5-8F cells and their corresponding controls. DEGs that met both the log_2_FC > 1 and *P* value < 0.05 criteria were identified (**Figure 4C**). Three groups of DEGs, upregulated genes in UPF1-KD 6-10B cells, upregulated genes in Emetine-treated 5-8F cells, and downregulated genes in UPF1-overexpressing HK1 cells, were considered potential NMD targets. A total of 212 DEGs simultaneously detected in two or three groups (**Supplementary Table 2**), and these DEGs were subjected to KEGG pathway enrichment analysis by using the WEB-based GEneSeT AnaLysis Toolkit (webgestalt) (https://www.webgestalt.org) on the basis of overrepresentation analysis (ORA). As shown in **Figure 4D**, these potential NMD targets were significantly enriched in multiple immune-inflammatory-related signaling pathways, including the NOD-like receptor signaling pathway, TNF signaling pathway, MAPK signaling pathway and IL-17 signaling pathway. To further validate the pathways associated with UPF1 in cancer, we utilized expression data from head and neck squamous cell carcinoma (HNSC) available in TCGA to conduct Pathway activity analysis based on MSigDB Hallmark gene sets. The results revealed that UPF1-low tumors exhibited significantly higher TNF-α/NF-κB, IL-6/JAK-STAT3, and Inflammatory Response signatures, consistent with our observations in NPC models (**Supplementary Figure 3A**).

### 3.4 COX-2 and PD-L1 were identified as NMD targets in NPC cells

As shown in **Figure 4C**, PTGS2 (a.k.a. COX-2), CD274 (a.k.a. PD-L1), CYP1B1, IFRD1, and AC135983.2 (a.k.a. WHAMMP1) were significantly upregulated in UPF1-KD and Emetine-treated NPC cells and downregulated in UPF1-overexpressing NPC cells, and their fold change in each group have been listed in **Supplementary Table 2**. COX-2 and PD-L1 are well known as critical regulators of cancer immune responses. Given the enrichment of UPF1-regulated genes in immune-related pathways, we focused on COX-2 and PD-L1 expression.

**Figure 5:**
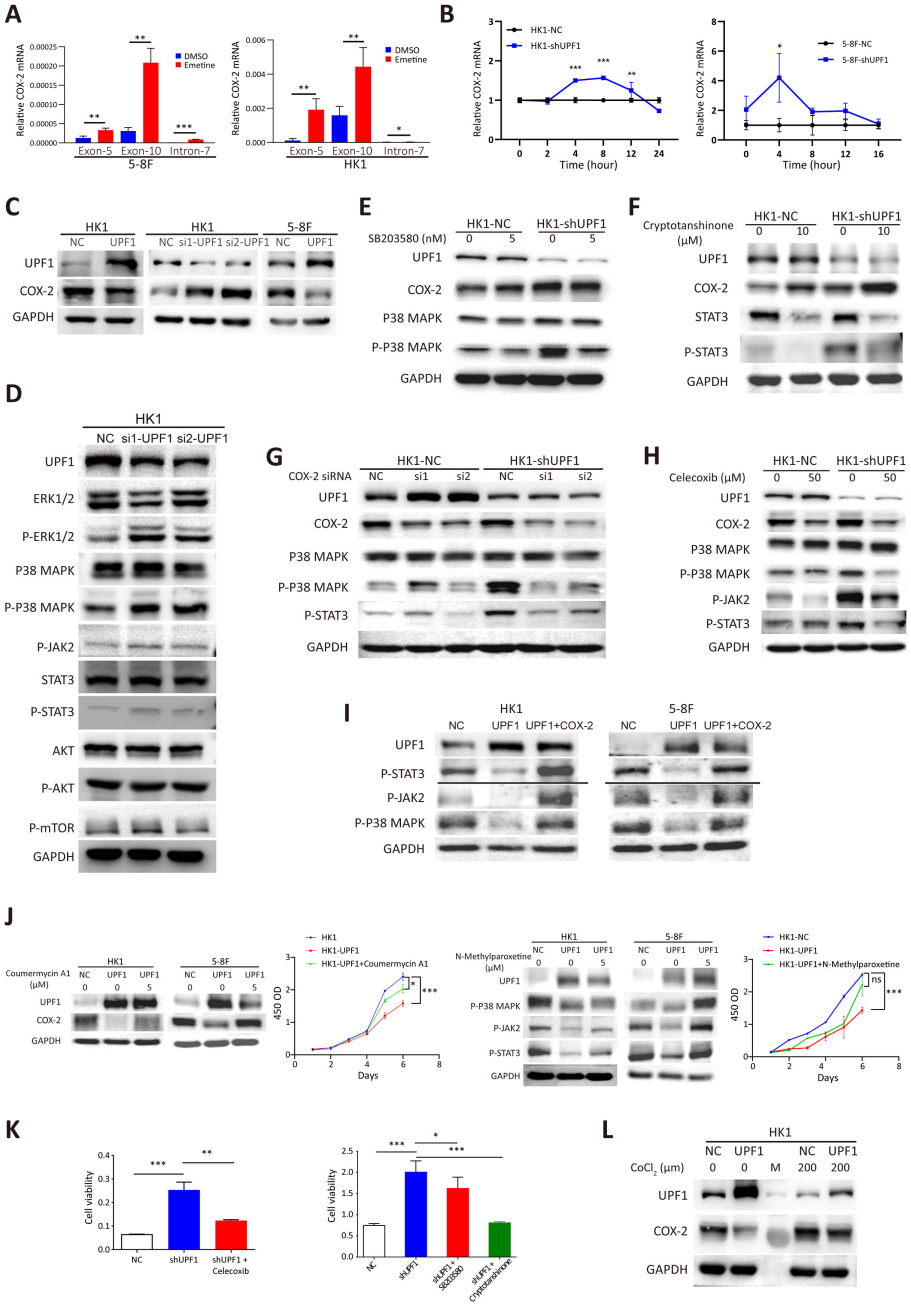
Reduced UPF1 enhances NPC cell growth through stabilizing COX-2 and activating the p38/MAPK and JAK2/STAT3 pathways. **(A)** COX-2 mRNA expression was elevated in Emetine-treated NPC cells, as determined by qRT–PCR. COX-2 mRNA was quantified by using primers from exon 5 or exon 10, and the alternative splicing transcript that retains intron 7 was quantified by using primers spanning the intron. The primers used are indicated on the X axis. **(B)** UPF1 knockdown by shRNA significantly increased the mRNA stability of COX-2 compared with that of the scrambled controls. Cells were pretreated with cycloheximide for 16 h, after which RNA was isolated at various time points following actinomycin D exposure. Transcript stability was determined by qRT–PCR. **(C)** Western blot analysis of COX-2 protein expression levels inUPF1-KD and UPF1-overexpressing NPC cells. **(D)** Phosphorylated MAPK and STAT3, but not mTOR, protein levels were increased in UPF1-KD NPC cells compared with those in control cells. **(E, F)** Upregulation of COX-2 protein expression in UPF1-KD cells was not attenuated by treatment with the p38 MAPK inhibitor SB203580 **(E)** or the STAT3 inhibitor cryptotanshinone **(F)**. **(G, H)** Activation of the p38/MAPK and JAK2/STAT3 pathways in UPF1-KD cells was abrogated by siRNAs against COX-2 **(G)** or the specific COX-2 inhibitor celecoxib **(H)**. **(I)** Transfection with a COX-2 expression vector rescued the inhibition of p38/MAPK and JAK2/STAT3 pathway activities resulting from UPF1 overexpression. **(J)** COX-2 and JAK2 activator Coumermycin A1 or MAPK activator N-Methylparoxetine restore COX-2 and MAPK activity, which were suppressed in UPF1-overexpressing HK1 and 5-8F cells. CCK-8 assay showed that the reactivation of the either COX-2 or MAPK pathway attenuated the growth-inhibitory effect of UPF1. **(K)** The results of the CCK-8 assay revealed that the increase in NPC cell viability induced by UPF1-KD was abolished by treatment with inhibitors of the COX-2, p38 MAPK or JAK2/STAT3 pathways. **(L)** UPF1 overexpression abrogated the increase in COX-2 protein expression induced by hypoxia in NPC cells. *, P < 0.05; **, P < 0.01; ***, P < 0.001.

Consistent with previous reports, our analysis revealed significantly upregulated COX-2 expression in tumor tissues relative to normal tissues across three GEO datasets (GSE12452, GSE34573, and GSE64634) (**Supplementary Figure 3B**). Previous Research has revealed two distinct mechanisms for COX-2 mRNA regulation: first, in breast cancer cells, alternative splicing that retains intron 7 between exons 7 and 8 targets the transcript for NMD; second, the long 3’-UTR harbors multiple AU-rich elements (AREs) within exon 10 that destabilize the COX-2 mRNA (17). To test the effect of NMD on COX-2 mRNA decay, qRT–PCR was used to quantify overall COX-2 mRNA levels by using primers from exon 5, the levels of variants retaining intron 7 were determined by using primers spanning intron 7, and the levels of long 3’-UTR by using primers from exon 10. Data in **Figure 5A** demonstrate that inhibition of NMD by emetine dramatically upregulated all three COX-2 transcript isoforms. Of these, the transcript containing the long 3’-UTR (amplified by the exon 10 primer) was the most abundant species under both basal and emetine-treated conditions, while the intron 7-retaining variant remained the least expressed. These data collectively indicate that the long 3’-UTR is a predominant determinant for NMD-mediated degradation of COX-2 mRNA. This post-transcriptional regulation mirrors a common physiological strategy, as approximately 10% of normal cellular mRNAs—particularly those with long 3’-UTRs—are NMD targets, allowing for swift adjustments in gene expression.

Next, a mRNA stability assay was performed to compare the degradation rates of COX-2 mRNA in response to UPF1 knockdown. The results revealed that UPF1 knockdown increased the stability of COX-2 mRNA transcripts (**Figure 5B**). Western blotting further confirmed that the protein levels of COX-2 were reduced in UPF1-overexpressing cells and increased in UPF1-KD cells (**Figure 5C**). Similar results were obtained for PD-L1 (**Supplementary Figures 4A-C**). These results suggest that COX-2 and PD-L1 are targets of UPF1-mediated NMD in NPC.

To verify the correlation between the expression of UPF1 and that of COX-2 and PD-L1 in NPC tissues, we performed a Pearson correlation analysis using the gene expression data obtained from GSE12452. As shown in **Supplementary Figure 4D**, a negative correlation was found between UPF1 and both COX-2 and PD-L1 expression. Moreover, UPF1 expression was negatively correlated with HIF-1α mRNA expression, as expected. Additionally, UPF1 mRNA expression level was negatively correlated with the co-inhibitory molecules HAVCR2/TIM-3 and CTLA-4, but positively correlated with the T-cell activation marker LAMP1/CD107.

### 3.5 Reduced UPF1 expression enhances cell growth through COX-2-mediated p38/MAPK and JAK2/STAT3 pathway activation in NPC cells

To investigate the molecular network affected by the dysregulation of UPF1, we detected the expression of the core components in the enriched KEGG pathways among the DEGs and the frequently activated pathways in NPC. The results revealed that UPF1-KD increased the protein expression of phosphorylated ERK/MAPK and STAT3 but had no obvious effect on the phosphorylation of AKT or mTOR (**Figure 5D**, **Supplementary Figure 5A**). In contrast, UPF1 overexpression attenuated signaling through the p38/MAPK and JAK2/STAT3 pathways (**Supplementary Figure 5B**).

Activation of the p38 MAPK and JAK2/STAT3 signaling pathways have both been reported to increase the expression level of COX-2 in cancers (25, 26), suggesting that UPF1 might regulate COX-2 expression by activating the p38 MAPK and JAK2/STAT3 pathways. However, the upregulation of COX-2 expression induced by UPF1-KD was not abrogated by pharmacological inhibitors against p38 MAPK or JAK2/STAT3 (**Figures 5E, F**).

On the other hand, p38 (27) and JAK2/STAT3 (28) have been reported to be downstream targets of COX-2. As shown in **Figures 5G, H**, the COX-2 inhibitor celecoxib or siRNAs against COX-2 markedly reversed the increase in p-p38 and p-STAT3 expression in UPF1-KD cells. In contrast, COX-2 inhibition in UPF1-NC cells had minimal effect on p38/MAPK and JAK2/STAT3 activity. This differential response suggests that COX-2 may exert its regulatory influence primarily when p38/MAPK and JAK2/STAT3 signaling are highly activated. In contrast, the suppressive effects of UPF1 overexpression on p38/MAPK and JAK2/STAT3 pathway activities were reversed by transfection with a COX-2 expression vector (**Figure 5I**). These results suggest that UPF1-KD might activate p38 and the JAK2/STAT3 signaling pathway through the upregulation of COX-2 expression in NPC cells.

To investigate the mechanism through which UPF1 influences NPC cell viability, rescue experiments were conducted using pharmacological activator or inhibitors targeting COX-2, p38 MAPK or the JAK2/STAT3 pathway. COX-2 and JAK2 activator Coumermycin A1 and MAPK activator N-Methylparoxetine were applied to restore COX-2 and MAPK activity, which were suppressed in UPF1-overexpressing cells. CCK-8 assay showed that the reactivation of either COX-2 or MAPK attenuated the growth-inhibitory effect of UPF1 (**Figure 5J**). On the other hand, the increase in NPC cell viability induced by UPF1-KD was abolished by treatment with these inhibitors, suggesting that UPF1-KD promoted NPC cell growth through the activation of the p38/MAPK and JAK2/STAT3 pathways, which was mediated by the upregulation of COX-2 expression (**Figure 5K**, **Supplementary Figure 5C**).

NMD is reportedly inactivated by the phosphorylation of eIF2α during hypoxia. Here, we showed that CoCl_2_-induced hypoxia in HK1 cells reduced UPF1 expression and increased COX-2 expression, whereas UPF1 overexpression abrogated these effects, indicating that NMD suppression mediated hypoxia-induced COX-2 upregulation (**Figure 5L**). Similar results were observed in PD-L1 (**Supplementary Figure 5D**).

Recent studies have shown that tumor-derived COX-2 and PD-L1 proteins are released into the culture medium and the circulation of cancer patients, where their levels correlate with tumor burden and prognosis (29–33). Based on these findings, we measured the concentrations of COX-2 and PD-L1 in NPC cell culture supernatants using ELISA. Our results demonstrated a significant increase in extracellular COX-2 and PD-L1 following UPF1 knockdown in NPC cells (Supplementary **Figure 5E**). These data suggest that UPF1-deficient NPC cells may enhance the release of COX-2 and PD-L1 into the extracellular milieu.

### 3.6 Coculture with UPF1-KD NPC cells promotes M2 macrophage polarization and migration

According to the results of the RNA-seq data analysis in this study, UPF1 target genes were enriched mainly in innate immune-related pathways. As the most abundant immune cells and central mediators of innate immune responses, macrophages play essential roles in microenvironment remodeling, cancer cell proliferation, metastasis and immunosuppression. COX-2 plays a critical role in regulating the immune response and macrophage differentiation. Therefore, we hypothesized that UPF1-KD might affect tumor-associated macrophage activation through COX-2.

**Figure 6:**
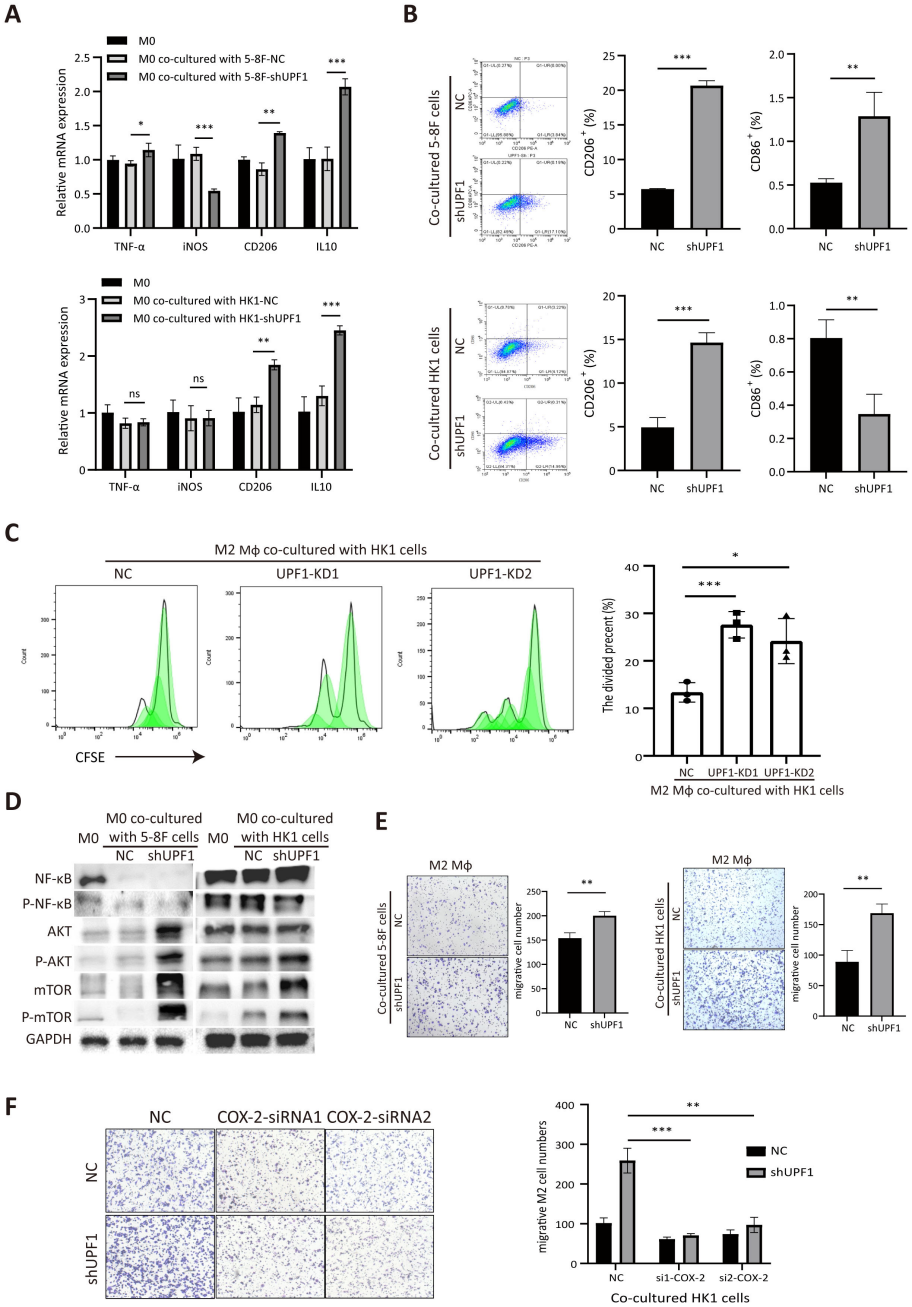
Coculture with UPF1-KD NPC cells promotes M2-type macrophage polarization and migration. **(A)** Polarization-specific biomarkers were analyzed by qRT–PCR using RNA collected from macrophages after coculture with NPC cells for 72 h. The M2 markers CD206 and IL10 were elevated in macrophages when they were cocultured with UPF1-KD cells. **(B)** Flow cytometry analysis revealed that the level of the M2 surface marker CD206 was significantly increased in macrophages cocultured with UPF1-KD NPC cells. **(C)** CFSE proliferation assays showed increase of M2 macrophage proliferation activity after cocultured with UPF1-KD cells compared with control. **(D)** Compared with those in control cells, the M2 macrophage pathway-related AKT/mTOR pathway in macrophages in which UPF1-KD NPC cells were cocultured was activated. **(E)** Compared with that of control cells, the migration rate of M2 macrophages was enhanced by coculture with UPF1-KD NPC cells. **(F)** COX-2 inhibition by siRNAs abrogated the increase in migration in UPF1-KD cells. *, P < 0.05; **, P < 0.01; ***, P < 0.001.

To investigate the impact of UPF1-KD NPC cells on macrophages, PMA-differentiated THP-1 cells were cocultured with UPF1-KD or control NPC cells by using a noncontact coculture system. M0 macrophages without coculture were used as the control. The qRT–PCR results revealed that M2-associated markers (CD206 and IL10) were significantly upregulated in M0 macrophages cocultured with UPF1-KD cells compared with those in control cells, whereas M1-associated markers (TNF-α and iNOS) were unchanged or decreased (**Figure 6A**). Flow cytometry analysis further confirmed the upregulation of the M2 marker CD206 in macrophages cocultured with UPF1-KD cells (**Figure 6B**). The M1 surface marker CD86 was decreased in macrophages cocultured with UPF1-KD HK1 cells but increased in those cocultured with UPF1-KD 5-8F cells (**Figure 6B**). Moreover, CFSE proliferation assays showed increase of M2 macrophage proliferation activity after cocultured with UPF1-KD cells compared with control (**Figure 6C**).

Furthermore, western blotting analysis revealed that the M2-related AKT/mTOR pathway was dramatically activated in macrophages cocultured with UPF1-KD NPC cells compared with that in control cells, while NF-κB phosphorylation, which contributes to macrophage M1 polarization, did not change (**Figure 6D**). These results suggested that UPF1 knockdown in NPC cells promotes macrophage differentiation toward the M2 phenotype.

To evaluate macrophage recruitment by NPC cells, we investigated the migration activity of M2 macrophages by using a Transwell insert with an 8 µm pore size. The results revealed that the migration rate was greater in M2 macrophages cocultured with UPF1-KD NPC cells than in those cocultured with control cells (**Figure 6E**).

To determine whether COX-2 mediated the effect of UPF1 on M2 macrophages, rescue experiments were performed by using siRNAs against COX-2 in NPC cells. The results showed that COX-2 inhibition in UPF1-KD NPC cells abrogated the increase in migratory macrophages after coculture, which indicated that the increase in macrophage recruitment enhanced by UPF1-KD was mediated by COX-2 (**Figure 6F**).

### 3.7 UPF1 deficiency in NPC cells inhibits CD8^+^ T-cell proliferation and activation

**Figure 7:**
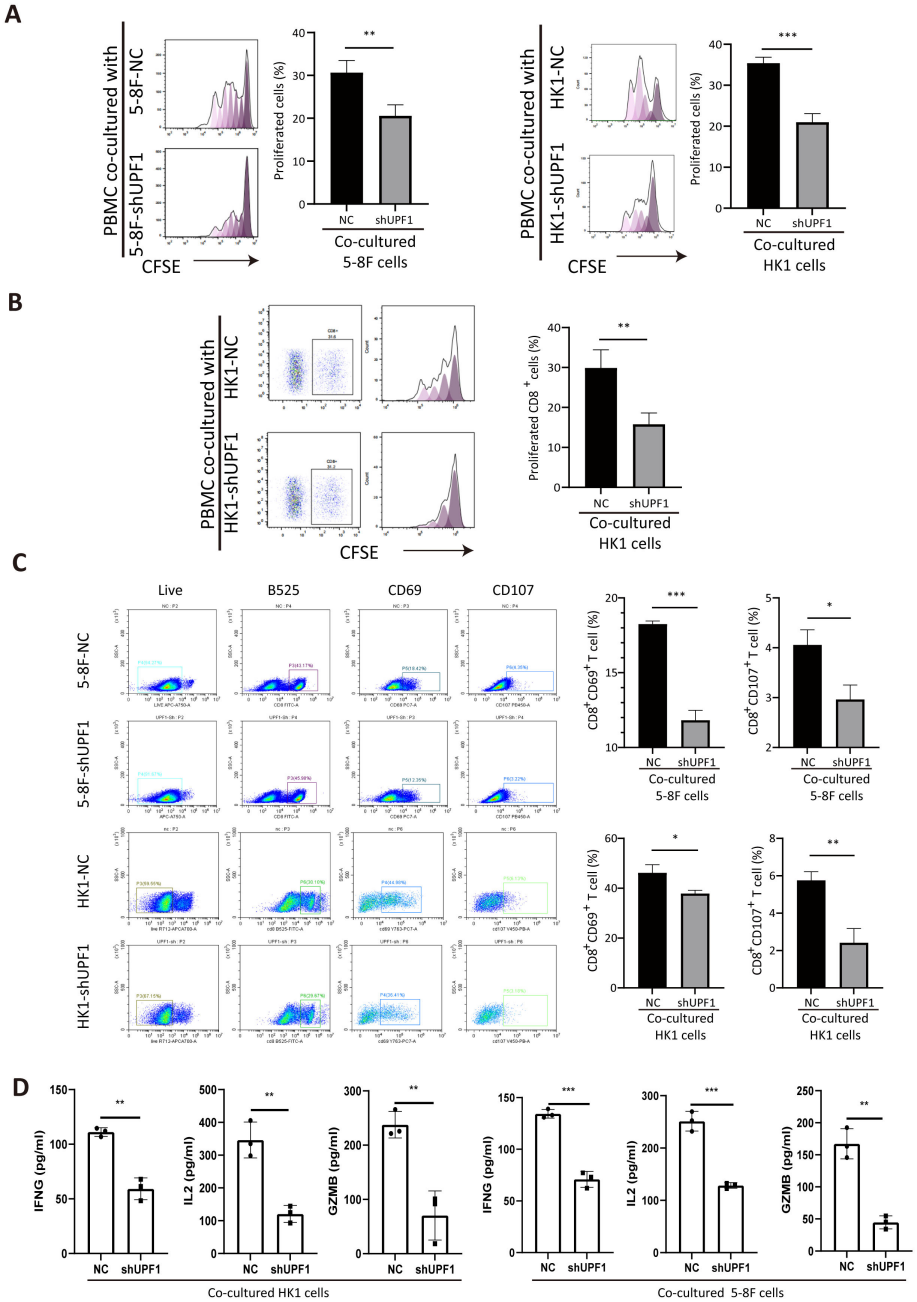
Coculture with UPF1-KD NPC cells inhibits CD8^+^ T-cell proliferation and activation. **(A)** Compared with control cells, anti-CD3/CD28-stimulated PBMCs cocultured with UPF1-KD NPC cells exhibited reduced proliferation ability, as assessed by a CFSE proliferation assay. **(B)** CFSE proliferation assays after gating on CD8^+^ cells revealed a significant decrease in the number of dividing CD8^+^ T cells after coculture with UPF1-KD NPC cells. **(C)** Flow cytometry analysis revealed that the percentages of CD8^+^CD69^+^ and CD8^+^CD107^+^ T cells significantly decreased after coculture with UPF1-KD NPC cells. **(D)** Following co-culture with UPF1-KD or control NPC cells, CD8^+^ T cell activity was assessed by measuring the secretion of IFNG, IL-2, and GZMB via ELISA. *, P < 0.05; **, P < 0.01; ***, P < 0.001.

To determine the impact of UPF1 on T-cell function, we cocultured anti-CD3/CD28-stimulated PBMCs with NPC cells. Compared with that of the control cells, the proliferation of the CFSE-labeled cells markedly decreased after 3 days of coculture with UPF1-KD NPC cells (**Figure 7A**). A similar reduction in the proportion of divided PBMCs was observed after gating on CD8^+^ cells (**Figure 7B**). Moreover, significant downregulation of the expression of the activation markers CD69 and CD107 in the CD8^+^ PBMC population was observed after coculture with UPF1-KD NPC cells, which was consistent with an inhibited phenotype in CD8^+^ T cells (**Figure 7C**). To further assess CD8^+^ T cell activity, we measured the production of IFNG, IL-2, and GZMB via ELISA after the T cells were co-cultured with UPF1-knockdown (KD) or control NPC cells. The results showed that exposure to UPF1-KD NPC cells significantly suppressed the production of these T cell activation markers (**Figure 7D**). These results suggested that coculture with UPF1-KD NPC cells might attenuate CD8^+^ T-cell activity.

## 4 Discussion

NPC is an epithelial malignancy that is prevalent in Southeast Asia and South China and is characterized by high metastasis and invasion potential. Approximately 70% of NPC patients are diagnosed at advanced stages because of its insidious behavior and nonspecific early symptoms (34). Although the survival rate of patients with early-stage NPC has increased with recent therapeutic advances, the overall survival of NPC patients is only 12–30 months once metastasis occurs (35, 36). Hence, elucidating the molecular mechanisms underlying the malignant progression of NPC is urgently needed.

Here, we verified that the expression level of UPF1, the core factor of the NMD pathway, was significantly lower both at the mRNA level and protein level in NPC tissues than in nontumor tissues and its low expression was correlated with a shorter survival time of NPC patients. UPF1 overexpression in NPC cells inhibited cell proliferation, migration, invasion and metastasis both *in vitro* and *in vivo*, whereas UPF1 knockdown resulted in the opposite effects. These results suggest that UPF1 plays a tumor-suppressing role in NPC cells.

Since the UPF1 expression level is critical for NMD activity, we hypothesized that the stabilization of NMD target genes as a consequence of impaired NMD in UPF1-KD cells might contribute to tumor progression. To screen for key UPF1 targets, RNA-seq was conducted by comparing UPF1-overexpressing, UPF1-KD, or Emetine-treated NPC cells with their controls. Analysis of RNA-seq data revealed that the potential UPF1 targets were enriched mainly in innate immune response-related pathways. COX-2, PD-L1, CYP1B1, IFRD1, and AC135983.2 were found to be simultaneously downregulated by UPF1 overexpression and upregulated by UPF1-KD or Emetine treatment. COX-2, PD-L1 and IFRD1 have been reported to be subject to NMD degradation in other cancers (17, 37, 38). Subbaram et al. (17) observed that the stability of COX-2 mRNA was increased by siRNA-mediated knockdown of UPF1, or treatment with NMD suppressing agent in breast cancer cells. CYP1B1 encodes a member of the cytochrome P450 superfamily of enzymes and can be induced by COX-2 in human breast cancer cells (39). Considering that the potential UPF1 targets were enriched mainly in immune-related signaling pathways, we focused on COX-2 and PD-L1, two crucial factors in immunomodulation and tumor progression, as candidate UPF1 targets in NPC.

First, an RNA stability assay confirmed that the transcripts of COX-2 and PD-L1 were stabilized by UPF1-KD. Western blotting analysis further demonstrated that the protein levels of COX-2 and PD-L1 were decreased by UPF1 overexpression but increased by UPF1 knockdown. These findings confirmed that COX-2 and PD-L1 mRNA were susceptible to UPF1-mediated degradation in NPC cells.

Both the mRNA and protein levels of COX-2 are reportedly elevated in tumor cells in NPC tissues and are associated with metastasis and an adverse prognosis (40–42). Moreover, the expression of COX-2 can be upregulated transcriptionally in response to hypoxia in cancers (43, 44). Our results showed that UPF1 overexpression could abrogate the increase in COX-2 expression induced by hypoxia in NPC cells, indicating that hypoxia induces COX-2 expression, at least in part, through NMD inhibition.

We further confirmed that both the ERK/MAPK signaling pathway and the JAK2/STAT3 signaling pathway were activated by UPF1-KD in NPC cells. Moreover, a rescue experiment revealed that this activation was dependent on the upregulation of COX-2 expression. Since the p38 MAPK and JAK2/STAT3 signaling pathways are known to be upstream regulators of COX-2 in cancer cells (26, 45), the activation of the ERK/MAPK and JAK2/STAT3 pathways by COX-2 may form a positive feedback loop to promote the survival of NPC cells. In the present study, we demonstrated that UPF1 deficiency enhanced NPC cell growth through the activation of the p38/MAPK and JAK2/STAT3 pathways, which was mediated by COX-2 upregulation.

The microenvironment of NPC is characterized by abundant immune cells shaped by chronic EBV infection and locoregional lymphoid infiltration. A higher density of CD163^+^ macrophages in NPC tissues, representing a protumoral M2 phenotype, was reported to predict worse survival (46, 47). CD3^+^ T lymphocytes are abundantly infiltrated into NPC tissues but lack an effective immune response (48). Transcriptomic studies have reported that CD8^+^ T-cell clusters in NPC are highly exhausted (49). It has been reported that hypoxic cancer cells induce M2 polarization through COX-2 (50). Xun et al. (51) reported that high levels of COX-2-expressing HCC cells can induce M2 macrophage polarization and activate CD8^+^ T-cell exhaustion. Moreover, we found that the extracellular concentration of COX-2 and PD-L1 proteins was elevated in the culture medium of UPF1-KD cells by using ELISA. Therefore, we hypothesized that UPF1-KD might affect tumor-associated macrophage activation through COX-2. Consistent with the findings of previous studies, macrophage M2 polarization increased after coculture with UPF1-KD NPC cells, as confirmed by the increased expression of the M2 markers CD206 and IL10, as well as the activation of the M2-related AKT/mTOR pathway. A rescue experiment confirmed that the recruitment of M2 macrophages by UPF1-KD NPC cells was mediated through COX-2. COX-2 is a potential therapeutic target in the treatment of NPC. Our data indicated that low UPF1 levels may predict sensitivity to COX-2-targeted therapy.

PD-L1, the dominant inhibitory ligand of PD-1 expressed on T lymphocytes, is upregulated on the surface of tumor cells in a broad range of cancer types, including NPC (52). Recent evidence has shown that PD-L1 overexpression can be triggered by hypoxia in various cell types in cancers (53–56). The possible mechanism might involve binding of HIF-1α to its promoter region or by cytokines (such as TNF-α and IFN-γ) produced in the hypoxic environment (57, 58). Here, we showed that PD-L1 mRNA stability and protein expression levels were significantly impacted by UPF1 expression and NMD activity. Moreover, UPF1 overexpression diminished the upregulation of PD-L1 under hypoxic conditions, indicating another mechanism of PD-L1 upregulation under hypoxic conditions as a consequence of NMD inhibition. NMD inhibition has been found to increase the proportion of aberrant transcripts and generate neoantigen candidates (1, 4), which might promote sensitivity to immunotherapy by enhancing immunogenicity. In our study, we revealed for the first time that UPF1 knockdown enhances PD-L1 expression, which may represent an additional mechanism by which UPF1 inhibition improves immunotherapy efficacy. Therefore, UPF1 may serve as a promising biomarker for predicting response to immunotherapy.

## 5 Conclusions

**Figure 8:**
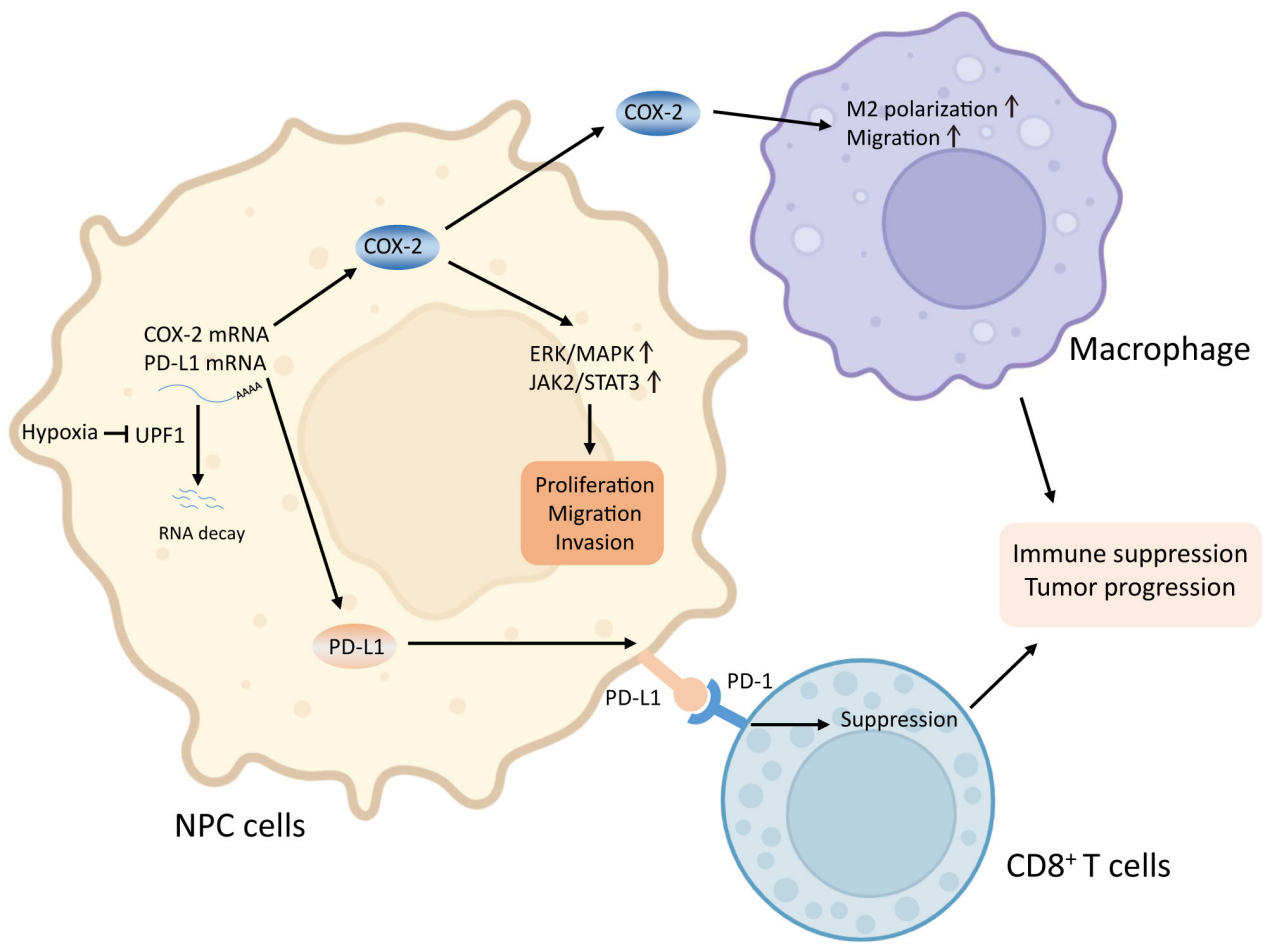
Graphical abstract. Reduced UPF1 expression in NPC impaired NMD efficiency, led to stabilization and upregulation of COX-2 and PD-L1, which activated the ERK/MAPK and JAK2/STAT3 pathways, promoted M2-switch of macrophages, and inactivated CD8^+^ T cell, thus enhanced NPC cell viability and metastasis.

In conclusion, our results suggest that hypoxia-induced suppression of UPF1 expression decreases NMD efficiency, leading to the stabilization of COX-2 and PD-L1 mRNAs, thus activating the p38/MAPK and JAK2/STAT3 pathways and promoting tumor progression in NPC cells. A mechanistic diagram is presented in **Figure 8**. Considering the general influence of NMD on gene expression and the prevalent upregulation of NMD targets in NPC, more NMD targets might be involved in malignant progression and need to be clarified in future studies.

## Data Availability

All sequencing data generated in the current study are available from the GEO repository with the accession number GSE305669 (https://www.ncbi.nlm.nih.gov/geo/query/acc.cgi?acc=GSE305669). Reference other datasets used and/or analyzed during the current study are available from the corresponding author on reasonable request.

## Glossary

NMD: nonsense-mediated mRNA decay
NPC: nasopharyngeal carcinoma
UPF1: upframeshift 1
COX-2: Cyclooxygenase-2
TAM: Tumor-associated macrophages
OS: overall survival
DFS: disease-free survival
NC: negative control
siRNA: small interfering RNA
shRNA: short hairpin RNA
KD: knockdown
qRT-PCR: quantitative real-time PCR
CFSE: carboxyfluorescein succinimidyl ester
IHC: immunohistochemistry
PMA: phorbol 12-myristate 13-acetate
Dox: doxycycline
ActD: Actinomycin D
TCGA: The Cancer Genome Atlas
GEO: Gene Expression Omnibus
webgestalt: WEB-based GEneSeT AnaLysis Toolkit
ORA: over-representation analysis
DEG: differentially expressed gene
