# Supplementary material for: Reduced expression of UPF1 promotes tumor progression through stabilizing COX-2 mRNA in nasopharyngeal carcinoma
Source: Front Immunol. 2025 Nov 10;16:1617864. doi: 10.3389/fimmu.2025.1617864 (PMC12640816; doi:10.3389/fimmu.2025.1617864)
Supplement: Supplementary file 1 [file DataSheet1.pdf]

Supplementary Figure 1

Multiplex immunohistochemistry (IHC) was performed on subcutaneous tumor biopsies obtained from mice injected with UPF1-NC and UPF1-KD HK1 cells. Two representative cases are presented: whole-slide images of H&E-stained sections are shown on the left. The black squares indicate the regions from which the high-magnification images on the right were taken. In these images, EPCAM-positive cells are stained brownish-red, while UPF1-positive cells are labeled green.

Supplementary Figure 2

Knockdown of UPF1 by siRNAs inhibit cell growth (**A**), colony formation (**B**) and migration (**C**) in 6-10B cells, and UPF1 overexpression exerts opposite effects in S18 cells.

Supplementary Figure 3

(A) Pathway analysis using neck squamous cell carcinoma (HNSC) expression data from TCGA showed UPF1-low tumors exhibited significantly elevated activity in the TNF-α/NF-κB, IL-6/JAK-STAT3, and inflammatory response pathways.

(B) Raw data from the GSE12452, GSE34573, and GSE64634 datasets analyzed by R(v4.5.1) revealed elevated PTGS2 expression and reduced UPF1 levels in tumor tissues compared to normal tissues.

Supplementary Figure 4

(A) PD-L1 mRNA expression was elevated in Emetine treated HK1 and 5-8F cells as determined by qRT-PCR.

(B) UPF1 knockdown by shRNA significantly increases mRNA stability of PD-L1 compared with scrambled controls, as determined by qRT-PCR.

(C) Western blot analysis of PD-L1 proteins was performed with protein extracted from UPF1-KD NPC cells.

(D) Correlation analysis between the mRNA expression of UPF1 and COX-2, PD-L1, HIF-1α, CD107, TIM-3 and CTLA-4 in NPC tissues in GSE12452

Supplementary Figure 5

(A) Phosphorylated MAPK and STAT3, but not mTOR proteins were increased in the UPF1-KD 6-10B cells compared with controls.

(B) UPF1 overexpression inhibits P38/MAPK and JAK2/STAT3 pathway activities in NPC cell lines by using western blot assay.

(C) The CCK-8 results showed that the enhanced 6-10B cell viability by UPF1-KD was abolished by treatment of the inhibitors against COX-2, p38 MAPK or JAK2/STAT3 pathway.

(D) UPF1 overexpression abrogated the increased PD-L1 proteins induced by hypoxia in NPC cells.

(E) The protein concentration of COX-2 and PD-L1 in culture supernatants from NPC-KD or control cells was detected by ELISA.
