# Supplementary material for: Reduced expression of UPF1 promotes tumor progression through stabilizing COX-2 mRNA in nasopharyngeal carcinoma
Source: Front Immunol. 2025 Nov 10;16:1617864. doi: 10.3389/fimmu.2025.1617864 (PMC12640816; doi:10.3389/fimmu.2025.1617864)
Supplement: Supplementary file 2 [file DataSheet2.pdf]

Supplementary table 1. Primer sequences used for qRT-PCR

**Genes**	Forward (5' - 3')	Reverse (5' - 3')
UPF1	CCTTCCCATCCAACATCTTC	AACATCGGTTTATCGGGTTG
NORM LacZA	GATGGCGTTAACTCGGCG	GCGAGGCGGTTTTCTCC
TER LacZB	GCTGTGCCGAAATGGTC	CCTGCC AGTATTTAGCG
COX-2-Exon-5	AGAAGAAAGTTCATCCCTGATCCC	AAAGCTGGCCCTCGCTTATGATCT
COX-2-Exon-10	TGCATTGGAATCAAGCCTGGCTAC	GGCAGAGTCCAAAGAAAGTGAACTC
COX-2-Intron7	TTGAGTAAATGACAAGATGTGG	CACAAGCTTTCAAGCAACTGG
PD-L1	TATGGTGGTGCCGACTACAA	TGCTTGTCCAGATGACTTCG
TNF-a	GAGGCCAAGCCCTGGTATG	CGGGCCGATTGATCTCAGC
iNOS	TTCAGTATCACAACCTCAGCAAG	TGGACCTGCAAGTTAAAATCCC
CD206	GGGTTGCTATCACTCTCTATGC	TTTCTTGTCTGTTGCCGTAGTT
IL10	GACTTTAAGGGTTACCTGGGTTG	TCACATGCGCCTTGATGTCTG
GAPDH	CTCCTCCTGTTCGACAGTCAGC	CCCAATACGACCAAATCCGTT

Supplementary table 2. Fold change of all the 212 overlapping DEGs screened from the RNA-seq results in this study (*P*<0.05)

	Up-regulated in UPF1-KD cells	Up-regulated in Emetine treated cells	Down-regulated in UPF1 overexpressing cells
Genes	log_2FC	log_2FC	log_2FC
**PTGS2**	1.83	2.60	-2.31
**CD274**	1.41	1.21	-1.82
**IFRD1**	1.27	1.03	-1.07
**CYP1B1**	1.09	1.83	-2.34
**AC135983.2**	1.17	1.03	-1.25
AC009121.2	3.56	5.64	
RGPD6	3.46	2.39	
NDUFA4L2	3.34	1.72	
KRT34	2.85	2.00	
TNF	2.39	5.57	
AL354696.1	2.36	2.91	
LCMT1-AS1	2.21	2.78	

ATF3	2.20	1.79
VN1R81P	2.14	1.89
AC027702.1	2.08	2.05
GPR3	2.06	2.47
CEP83-AS1	1.96	2.52
AC026471.6	1.95	2.78
CTGF	1.95	2.37
AL645608.8	1.92	1.48
MPZL2	1.88	1.07
AL133216.2	1.81	1.55
FST	1.78	1.70
AC004471.1	1.75	2.48
WFDC21P	1.74	1.51
COL4A4	1.73	5.64
CGA	1.69	2.26
ASNS	1.65	2.86
AC103706.1	1.62	1.53
SNHG7	1.59	1.76
AL669831.3	1.57	2.28
AC096733.2	1.57	1.55
CCL2	1.56	1.53
HOXB9	1.53	1.47
CSF2RA	1.50	2.11
CYR61	1.47	1.77
CLDN1	1.46	1.73
STAG3L4	1.41	1.39
HBEGF	1.40	1.08
CU633906.2	1.39	3.22
AC093673.1	1.39	1.33
CHMP4C	1.38	1.06
C8orf4	1.37	2.78
MAFF	1.34	3.27
SGK1	1.33	1.46
AC112907.2	1.31	1.19
RGPD1	1.30	2.78
DLGAP1-AS2	1.30	1.58
AL512488.1	1.24	2.21
CU639417.2	1.24	1.89
PLAU	1.21	2.07
ZFPM2-AS1	1.20	1.49

FOSB	1.16	2.65	
CPLX1	1.15	1.03	
NUPR1	1.13	4.11	
ADM2	1.13	3.24	
DUSP13	1.11	1.53	
STMN3	1.11	2.11	
TM4SF1	1.09	1.36	
HES4	1.09	1.89	
SEMA7A	1.08	1.66	
DUSP5	1.07	3.08	
TNFRSF9	1.06	2.37	
RGS16	1.06	1.26	
DUSP1	1.02	2.23	
NUP62CL	1.01	1.13	
MAPK10	7.29		-1.62
TC2N	6.87		-2.08
AL023284.4	6.29		-4.34
AC016747.3	5.79		-4.45
AKAP6	3.46		-2.30
CBWD4P	3.08		-2.88
AC006042.3	2.67		-4.82
ABCA11P	2.52		-2.25
GXYLT1	2.25		-1.56
AL117209.1	2.04		-2.49
PRRG4	1.96		-1.61
ALMS1-IT1	1.93		-2.58
CCDC121	1.88		-1.63
ZNF678	1.77		-1.99
SETD7	1.68		-1.50
SULF1	1.62		-1.48
CALD1	1.60		-1.79
GBP1	1.58		-1.44
PRKG2	1.57		-2.24
PIH1D2	1.56		-1.45
ZNF165	1.52		-1.74
AL161891.1	1.49		-1.46
FGD4	1.48		-2.74
MAP3K7CL	1.48		-1.55
ZNF430	1.48		-2.14
ZNF551	1.46		-1.19

GCSAM	1.46	-1.73
PLCB4	1.44	-1.34
RBL1	1.41	-1.86
CSNK1G1	1.41	-1.60
ICA1L	1.40	-1.96
ZNF664	1.40	-1.54
ZNF680	1.39	-1.55
G3BP2	1.34	-1.55
ZNF367	1.31	-1.50
KCNAB1	1.27	-3.03
ZW10	1.27	-2.04
MPP7	1.27	-2.29
DIAPH3	1.25	-2.09
FKBP14	1.23	-1.29
SRGAP2B	1.22	-1.87
DCP2	1.21	-1.97
ZNF714	1.20	-5.43
CDKN2AIP	1.17	-1.23
PLCXD2	1.15	-1.20
NOSTRIN	1.14	-2.62
EXPH5	1.14	-2.32
LPP	1.12	-2.36
KITLG	1.12	-2.66
WBP4	1.12	-1.02
VGLL3	1.11	-2.31
ZNF227	1.11	-1.30
CCDC150	1.11	-1.73
DNAJC25	1.10	-1.34
RAD51AP1	1.08	-1.47
AC060766.1	1.08	-2.48
RELL1	1.06	-1.15
ATAD1	1.05	-1.67
SLC5A3	1.05	-1.65
ATAD5	1.05	-2.09
UBE2V1	1.04	-1.57
SYT1	1.04	-1.69
CLIP1	1.04	-2.38
BLOC1S6	1.02	-1.93
TTC33	1.02	-1.61
CEP128	1.01	-1.85

CAVIN4	5.64	-1.96
DDIT3	3.63	-1.03
AC018809.1	3.45	-3.10
RBM44	3.45	-2.75
EREG	3.45	-1.56
AC013394.1	2.78	-2.02
TRPC1	2.77	-1.61
LINC01363	2.42	-4.17
SLITRK6	2.42	-1.08
SMPDL3A	2.23	-1.79
AL137003.2	2.21	-2.82
AL021368.2	2.08	-2.36
CFAP69	2.05	-3.11
GOLGA6L10	2.03	-3.11
ZC3H6	1.93	-2.10
C9orf72	1.82	-1.90
SHC4	1.74	-2.85
TMEM154	1.72	-1.36
ITGA2	1.70	-1.73
CD44-AS1	1.67	-2.63
AL137782.1	1.63	-1.86
KLHL24	1.61	-2.82
CCNG2	1.57	-1.56
ATP6AP2	1.56	-1.30
CCNL1	1.55	-1.88
SCML1	1.52	-1.80
PHLDB2	1.52	-2.39
TMOD2	1.52	-1.71
ADRB1	1.52	-1.13
YPEL5	1.49	-1.14
SLC38A2	1.49	-2.08
CREBRF	1.48	-2.96
CAPRIN2	1.48	-1.84
MCTP2	1.47	-1.68
TBC1D32	1.47	-2.23
PPP4R1L	1.44	-1.02
TCP11L2	1.41	-1.72
HMGCS1	1.38	-1.27
KLF7	1.37	-1.58
AL390728.4	1.36	-1.31

MMD	1.34	-1.16
MXD1	1.33	-1.99
GBP3	1.32	-2.21
RND3	1.32	-1.80
CLK1	1.30	-2.12
IL1RAP	1.29	-1.75
KLF3	1.28	-1.33
SLC7A11	1.28	-1.83
AC005839.1	1.27	-1.99
HIST2H2BE	1.26	-1.45
CDC14A	1.25	-2.06
TRMT11	1.24	-1.21
SLFN5	1.21	-2.06
CCT6P1	1.20	-1.70
PI4KAP1	1.19	-1.19
BIRC3	1.15	-2.07
RABGGTB	1.15	-1.30
B3GNT5	1.14	-2.45
CASP1	1.12	-1.58
C3	1.11	-1.09
COQ10B	1.11	-1.27
NT5E	1.11	-1.13
UBR5-AS1	1.09	-1.89
CASP4	1.09	-1.02
MEX3C	1.08	-1.39
CLDND1	1.08	-1.62
AC110079.1	1.08	-1.14
CD55	1.07	-1.34
ZNF75D	1.07	-1.16
FAS	1.06	-1.41
TAF1D	1.06	-1.51
WDR19	1.05	-1.87
HMGCR	1.05	-1.38
ZBTB26	1.03	-1.72
GOLGA6L9	1.03	-2.46
NAV3	1.02	-1.80
KLF9	1.01	-1.98
ASAP2	1.01	-1.26
CLIP4	1.01	-1.21
ARFGAP3	1.00	-1.11
