# Supplementary figures and images for: Reduced expression of UPF1 promotes tumor progression through stabilizing COX-2 mRNA in nasopharyngeal carcinoma
Source: Front Immunol. 2025 Nov 10;16:1617864. doi: 10.3389/fimmu.2025.1617864 (PMC12640816; doi:10.3389/fimmu.2025.1617864)

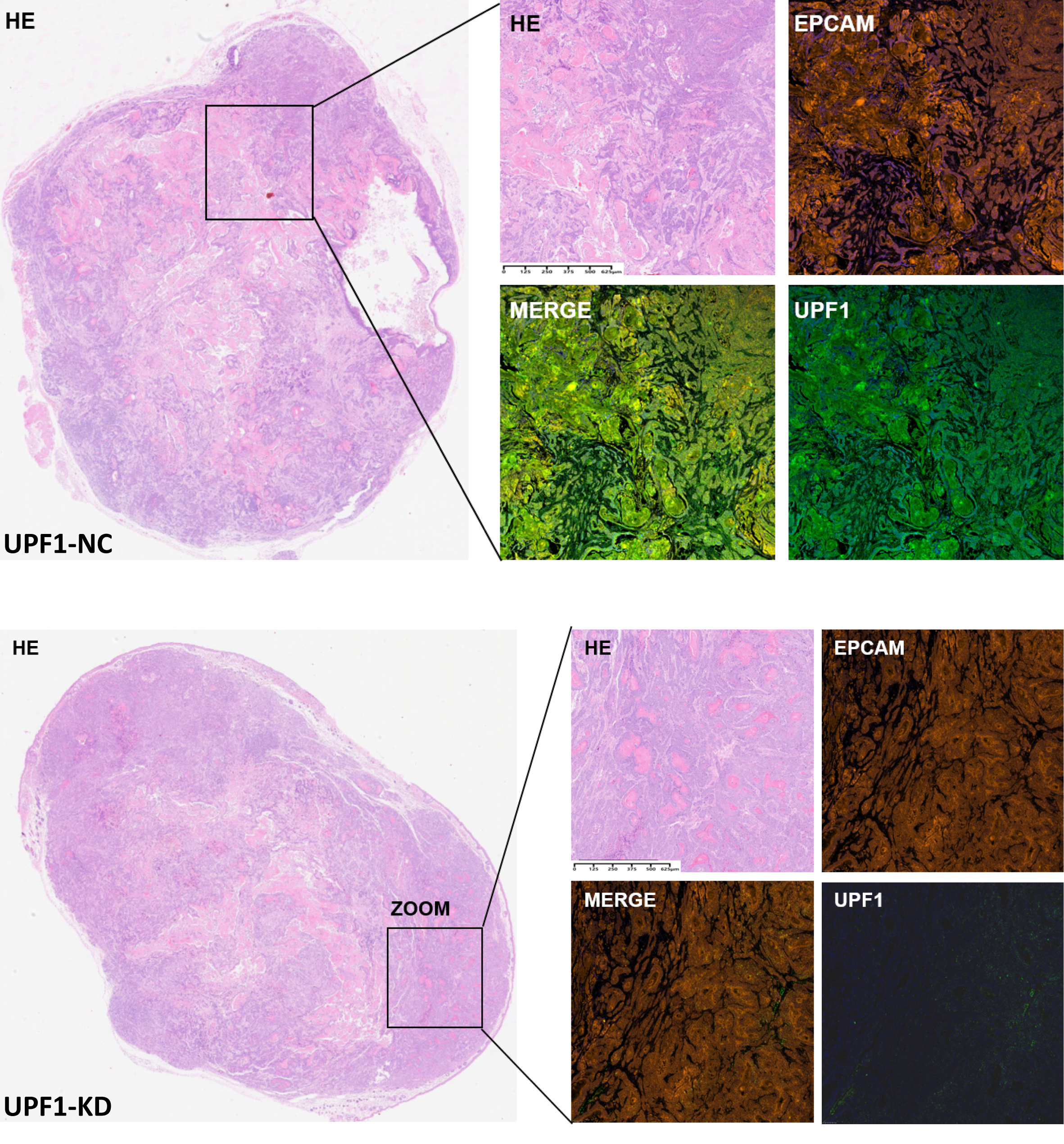

Supplement: Supplementary file 3 [file Image1.tif]

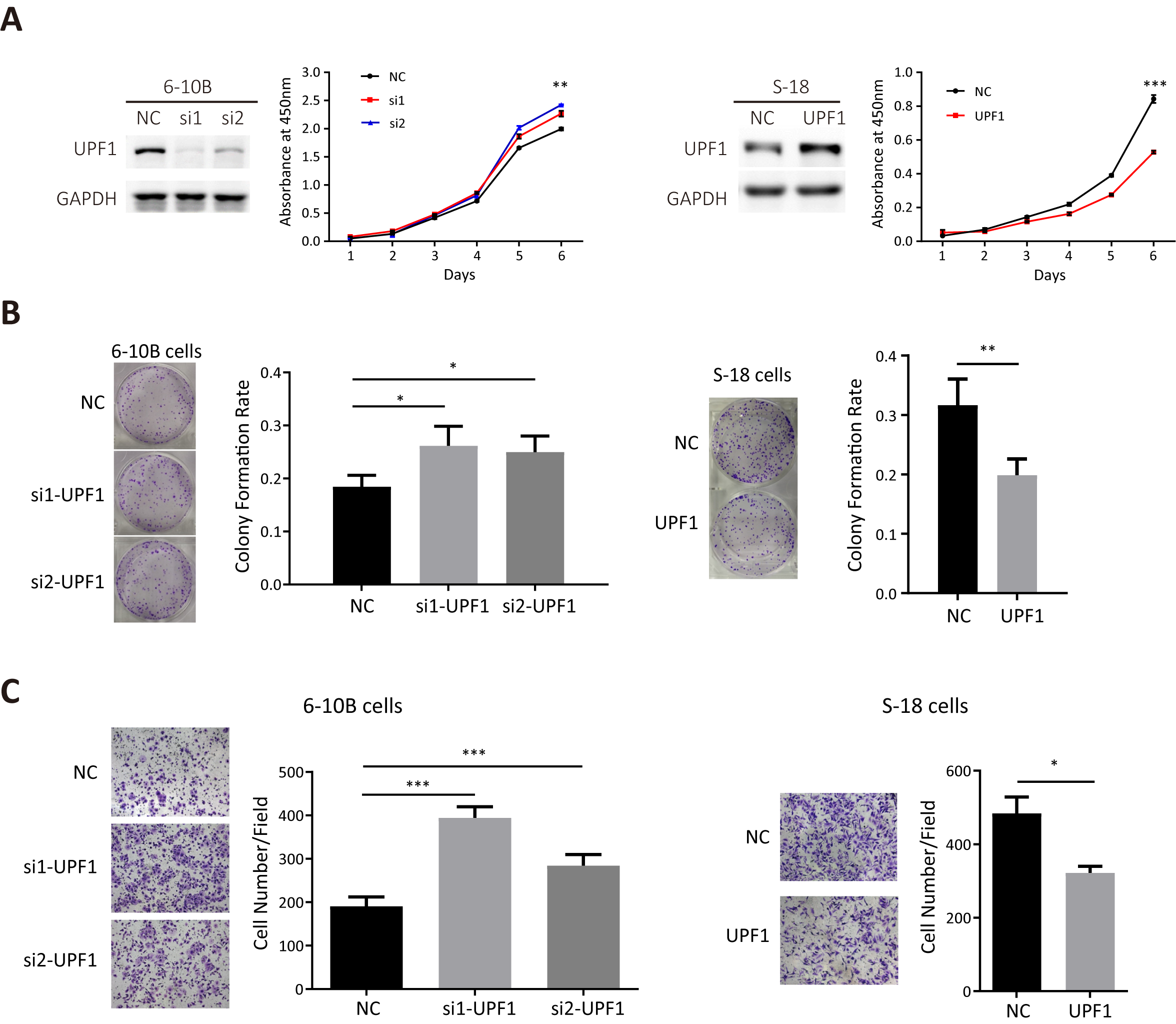

Supplement: Supplementary file 4 [file Image2.tif]

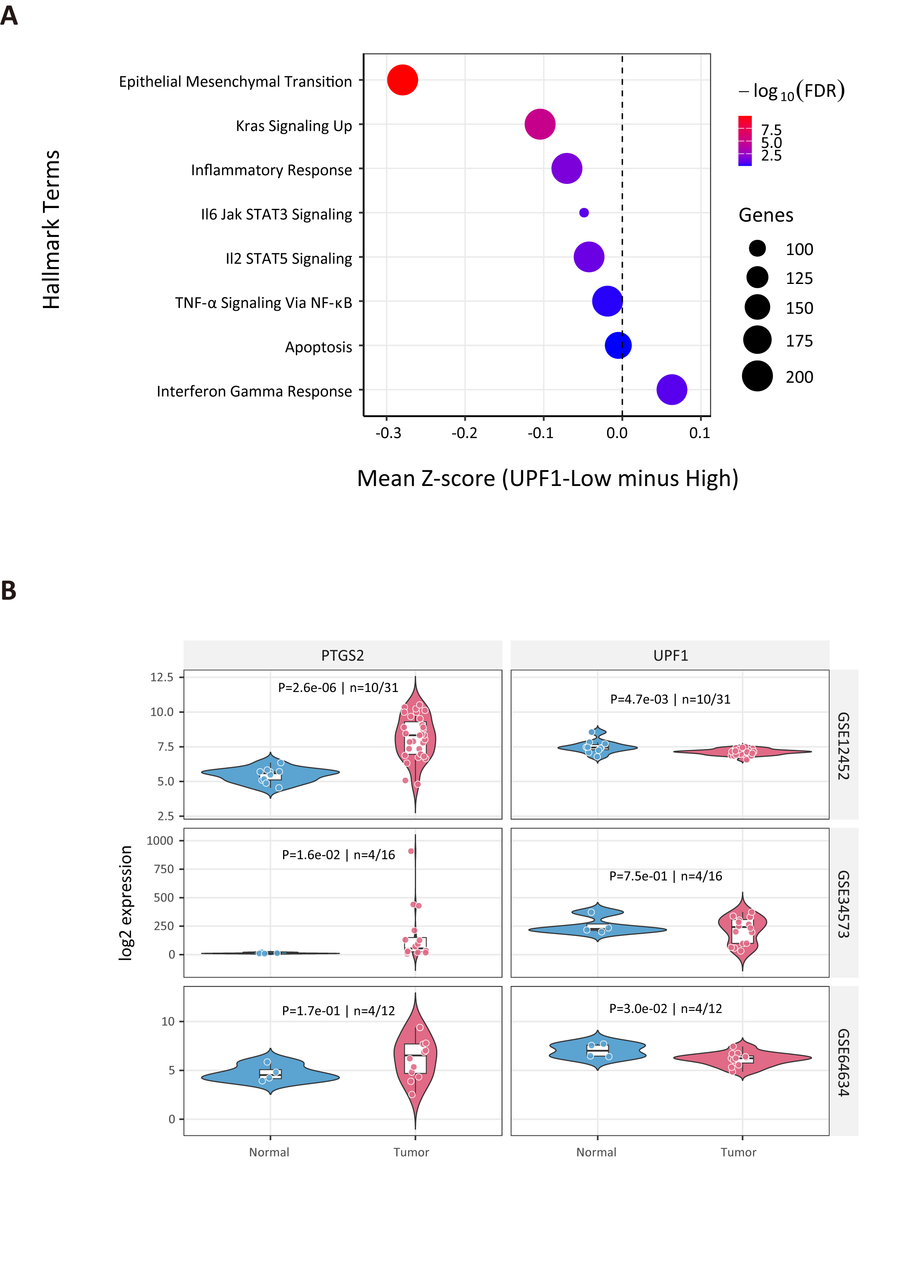

Supplement: Supplementary file 5 [file Image3.tif]

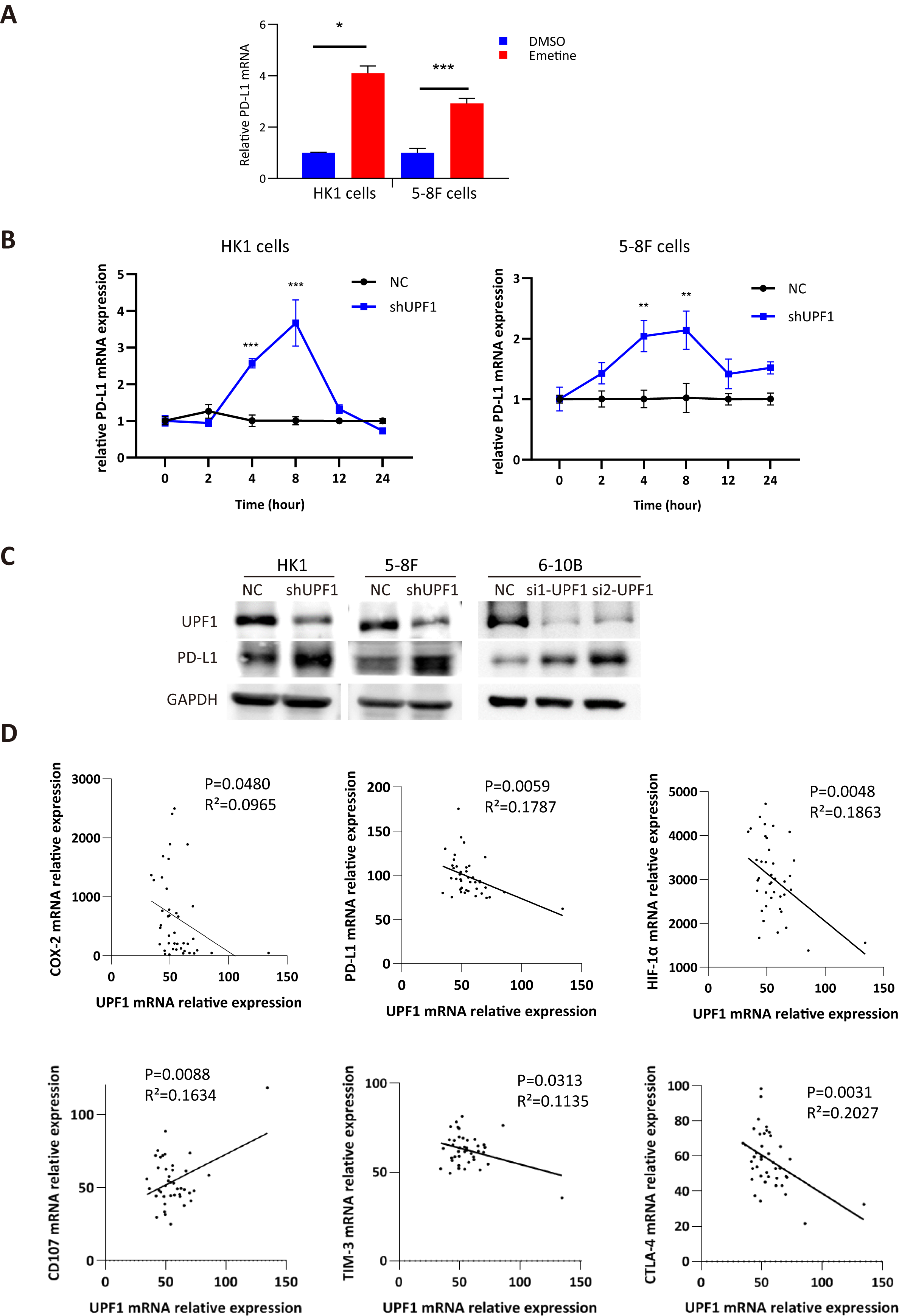

Supplement: Supplementary file 6 [file Image4.tif]

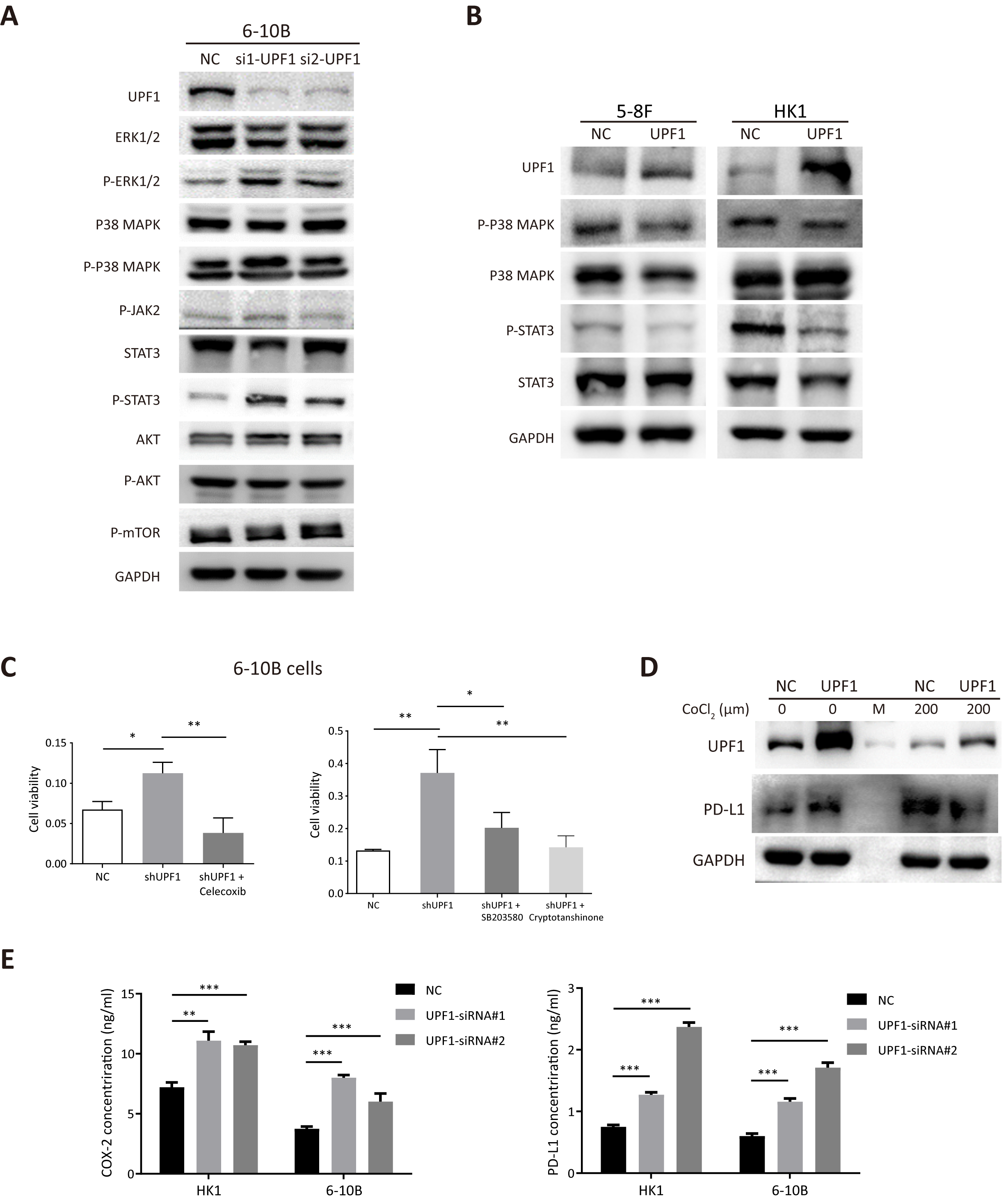

Supplement: Supplementary file 7 [file Image5.tif]
